# Quantum tunnelling and charge accumulation in organic ferroelectric memory diodes

**DOI:** 10.1038/ncomms15841

**Published:** 2017-06-12

**Authors:** Matteo Ghittorelli, Thomas Lenz, Hamed Sharifi Dehsari, Dong Zhao, Kamal Asadi, Paul W. M. Blom, Zsolt M. Kovács-Vajna, Dago M. de Leeuw, Fabrizio Torricelli

**Affiliations:** 1 Department of Information Engineering, University of Brescia, Via Branze 38, 25123 Brescia, Italy; 2 Max Planck Institute for Polymer Research, Ackermannweg 10, 55128 Mainz, Germany; 3 Graduate School Materials Science in Mainz, Staudinger Weg 9, 55128 Mainz, Germany; 4 Faculty of Aerospace Engineering, Delft University of Technology, Kluyverweg 1, 2629 HS Delft, The Netherlands

## Abstract

Non-volatile memories—providing the information storage functionality—are crucial circuit components. Solution-processed organic ferroelectric memory diodes are the non-volatile memory candidate for flexible electronics, as witnessed by the industrial demonstration of a 1 kbit reconfigurable memory fabricated on a plastic foil. Further progress, however, is limited owing to the lack of understanding of the device physics, which is required for the technological implementation of high-density arrays. Here we show that ferroelectric diodes operate as vertical field-effect transistors at the pinch-off. The tunnelling injection and charge accumulation are the fundamental mechanisms governing the device operation. Surprisingly, thermionic emission can be disregarded and the on-state current is not space charge limited. The proposed model explains and unifies a wide range of experiments, provides important design rules for the implementation of organic ferroelectric memory diodes and predicts an ultimate theoretical array density of up to 10^12^ bit cm^−2^.

## Abstract (web summary)

Organic non-volatile memories based on ferroelectric and semiconductor polymers are one of promising candidates for flexible electronics, yet the relevant device physics remains elusive. Ghittorelli *et al*. show that quantum tunnelling and charge accumulation govern the ferroelectric memory operation.

Non-volatile memories—providing the information storage functionality—are crucial circuit components, finding application in several fields such as health care, wellness, communication, automotive, entertainment, consumer electronics, and so on^1^,^2^,^3^. Solution-processed organic non-volatile memory diodes based on ferroelectric and semiconducting polymers are recognized as the non-volatile memory candidate for flexible electronics^4^. Organic ferroelectric memory diodes comprise blends of a semiconducting polymer and the ferroelectric copolymer of vinylidenefluoride with trifluoroethylene (P(VDF-TrFE))^5^,^6^,^7^,^8^,^9^,^10^,^11^,^12^,^13^,^14^,^15^,^16^,^17^,^18^. Upon film formation, the blend decomposes by spinodal phase separation yielding a microstructure that consists of bicontinuous columnar domains of the semiconducting polymer embedded in the matrix of P(VDF-TrFE)^12^,^13^,^16^,^19^. In the memory diode the blend film is sandwiched between two electrodes. The injecting electrode is deliberately chosen such that it forms a high-barrier Schottky contact with the semiconductor. Hence, the charge transport is injection limited and the current density is low; the diode is in the off-state. When the ferroelectric polymer is fully polarized, the current density is high and the diode is in the on-state. The diode can reversibly be switched by reversing the ferroelectric polarization. A 9 bit (3 × 3) cross-bar memory array was demonstrated in 2010 for the first time^20^. State of the art is the industrial demonstration of a 1 kbit reconfigurable array fabricated on plastic foil^4^.

Further progress, however, is limited owing to the lack of understanding of the device physics, which is required for the technological implementation of high-density arrays. Originally, it was suggested that the ferroelectric polarization leads to band bending in the semiconductor at the injecting contact^5^,^6^,^18^,^21^,^22^. This explanation however required a particular three-dimensional morphology showing an undercut of the ferroelectric slab, which was not observed in thorough morphological analyses^12^,^13^,^16^,^19^. In fact, the driving force for resistive switching is the modulation of the injection barrier by the ferroelectric polarization. Later, it was proposed, basing on numerical simulations, that the origin of the barrier lowering is the stray electric field between the polarization charges of the ferroelectric polymer and the compensating image charges in the electrode^8^,^9^. The ferroelectric polarization has been artificially modelled by means of fixed charges located at 1.5 nm distance from the electrode. The calculated current modulation was rationalized as a function of injection barrier. However, the complete current–voltage (*I–V*) curves could not be modelled because the polarization was simply described by fixed charges, disregarding its dependence on the electric field.

Here, we perform two-dimensional (2D) numerical simulations including the 2D polarization of the ferroelectric polymer, the charge injection at the metal–semiconductor interface and the charge transport in the organic semiconductor. We show that the full *I–V* characteristics as a function of both bias and temperature can be quantitatively modelled. The simulations reveal the crucial role of lateral ferroelectric polarization on the charge transport. The charge injection is analysed and quantum tunnelling is identified as the dominant mechanism. Finally, the interfacial charge transport and the consequences for downscaling are discussed.

## Results

### Structure and electrical characteristics of memory diodes

**Figure 1:**
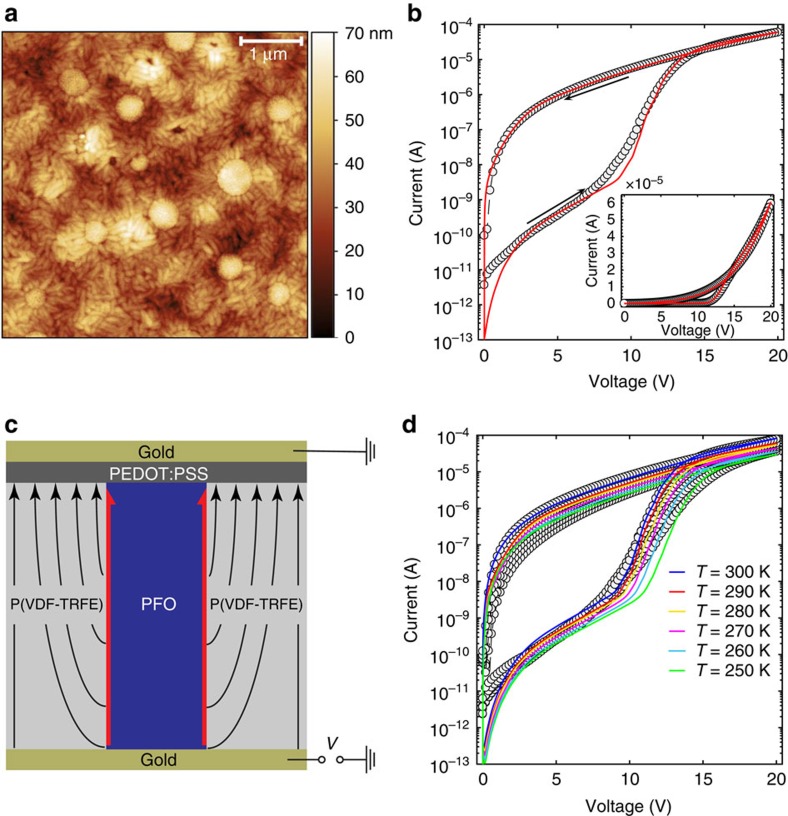
Structure and characteristics of organic ferroelectric memory diodes. (**a**) AFM micrograph of a phase-separated blend film of PFO and P(VDF-TrFE). The film thickness amounted to 265±10 nm. (**b**) Schematic cross-section of a ferroelectric memory diode. For the sake of clarity, here only one pillar of PFO surrounded by P(VDF-TrFE) is shown. The black arrows indicate the 2D polarization of the ferroelectric polymer. Holes are efficiently transported at the PFO/P(VDF-TRFE) interface (red arrows). (**c**) Measured (symbols) and simulated (line) *I–V* characteristics at ambient temperature (290 K). (**d**) Measured (symbols) and simulated (lines) *I–V* characteristics as a function of temperature.

Phase separated blend memory diodes were fabricated using the ferroelectric polymer P(VDF-TrFE) and the semiconducting polymer poly(9,9-dioctylfluorene) (PFO) by means of wire-bar coating, according to the method proposed in ref. 12. Further details are reported in the Methods section. The device area amounted to 0.16 mm^2^. An atomic force microscopy (AFM) height image is presented in Fig. 1a. The microstructure exhibits the characteristic morphology of a phase separated blend and consists of bicontinuous columnar PFO domains embedded in a P(VDF-TrFE) matrix^12^. The needle-like morphology is typical for semicrystalline P(VDF-TrFE)^23^,^24^. The schematic cross-section of a memory diode is shown in Fig. 1b. This microstructure is used as device geometry for 2D numerical simulations. The *I–V* characteristics measured at ambient temperature in dynamic vacuum of 10^−6^ mbar are presented in Fig. 1c (symbols). The top PEDOT:PSS contact is grounded and we sweep the bottom Au electrode from 0 to +20 V and back. We start in the off-state. At low bias the current density is low as the charge injection at the Au-PFO contact is limited. By increasing the positive bias, the ferroelectric P(VDF-TrFE) gets fully polarized above the coercive voltage, here about 10 V. The current then increases by orders of magnitude. Upon sweeping back to 0 V, the ferroelectric polarization does not change and the diode remains in the on-state. To switch off the diode, the ferroelectric polarization has to be reversed by applying a negative voltage larger than the coercive voltage.

### Numerical model

To explain the operation of ferroelectric memory diodes we reproduced the measured electrical characteristics as a function of temperature by means of 2D numerical simulations. The charge transport in the organic semiconductor is described by means of the variable range hopping theory^25^ and accounting for the disorder^26^,^27^,^28^. A Gaussian density of states centred at the Highest Occupied Molecular Orbital (HOMO) level of PFO is assumed^29^,^30^,^31^. The Poisson, continuity and drift-diffusion transport equations are solved on a 2D grid^32^,^33^,^34^,^35^. The charge flow at the metal–semiconductor contact is described accounting for the drift-diffusion, energy disorder, thermionic emission, tunnelling and image force barrier lowering^36^,^37^,^38^,^39^,^40^. The numerical simulations account for the 2D energy barrier, electric field and charge concentration. We emphasize that the polarization of the ferroelectric polymer as a function of the electric field is explicitly taken into account^34^,^41^,^42^. The charge flow at the metal–semiconductor contacts, the charge transport in the semiconductor, and the ferroelectric polarization are solved together on a 2D grid that accounts for the device geometry. The numerical framework and the physical model are detailed in the Supplementary Notes 1–4.

The model parameters are the following. The transport parameters of the semiconductor, namely the average intersite distance *d*_a_=1.5 nm and the energy disorder *σ*=0.16 eV, are obtained by modelling the temperature-dependent measurements of a PFO hole-only diode with Ohmic contacts (Supplementary Fig. 1). The HOMO level of PFO is taken as *E*_HOMO_=5.8 eV (refs 43, 44). Since the Au work function is taken as 4.5 eV (refs 45, 46), the contact barrier is Φ_B0_=1.3 eV (ref. 6). We extracted the ferroelectric parameters of P(VDF-TrFE), namely the relative permittivity, the remanent polarization, the saturation polarization and the coercive field as a function of temperature from thin film capacitor measurements (Supplementary Fig. 2). All the model parameters are listed in Supplementary Table 1. The diode thickness is 265±10 nm and the areal density of PFO pillars is estimated from AFM topography analysis. Since the diameter of the PFO pillars randomly varies along the area of the device, we measured the topography on several spots and we found that the average PFO/P(VDF-TrFE) interface length is 16.8±2.3 μm on an area of 5 × 5 μm^2^, as shown in Supplementary Note 5. The measured and calculated *I–V* characteristics at ambient temperature are shown on a semilogarithmic scale in Fig. 1c. The inset shows the *I–V* characteristics on a linear scale. The temperature-dependent curves are presented in Fig. 1d. The simulations nicely predict both the on-state and the off-state current in the whole range of biases and temperatures.

### Operation of memory diodes

**Figure 2:**
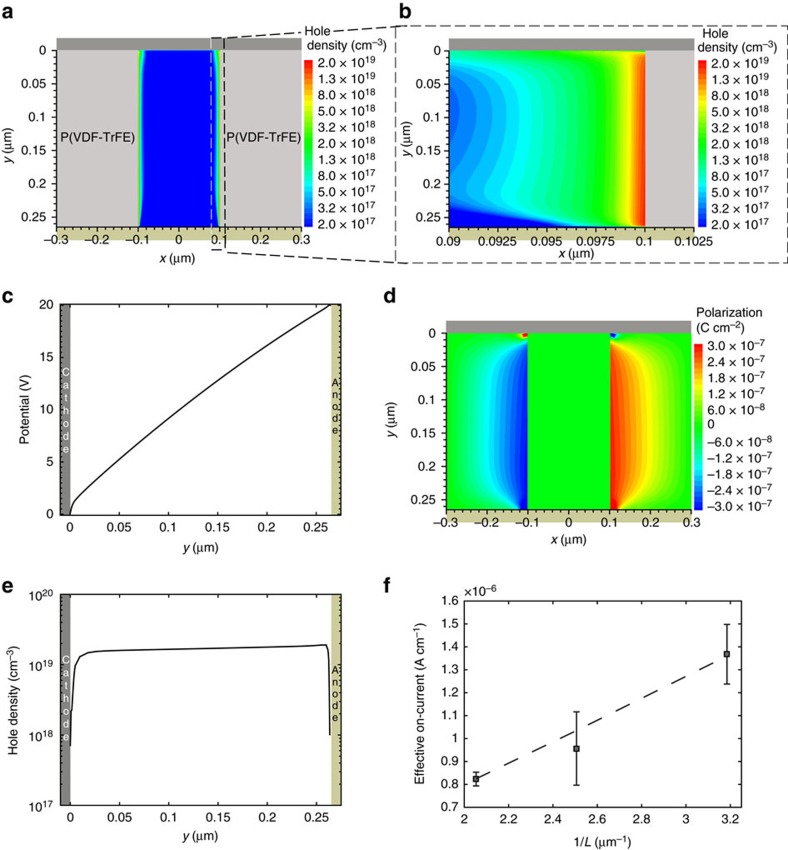
Operation of organic ferroelectric memory diodes. (**a**) Hole density distribution in the PFO pillar at *V*=20 V. (**b**) Zoom of the hole density distribution at the right PFO/P(VDF-TrFE) interface. (**c**) Potential along the PFO/P(VDF-TrFE) interface (cut line at *x*=0.099 μm), *V*=20 V. The anode corresponds to the injecting contact, while the cathode corresponds to the collecting contact. (**d**) *x-*component of the polarization vector at *V*=20 V. (**e**) Hole density along the PFO/P(VDF-TrFE) interface (cut line at *x*=0.099 μm), *V*=20 V. (**f**) Effective (viz. interface length normalized) current at *V*=10 V as a function of the inverse of the device thickness (*L*). The dashed line is the linear least-square approximation. The error bars are the s.d.

The 2D numerical simulations provide physical insight into the device operation. By increasing the applied voltage, the ferroelectric polymer polarizes and, owing to the stray field, the charge carriers are efficiently injected into the semiconductor. Figure 2a shows the distribution of the charge density in the PFO pillar when biased at 20 V. The injected charge carriers are located at the ferroelectric–semiconductor interfaces along the whole thickness of the diode. Two separated ‘channels' are formed. Figure 2b is a zoom of Fig. 2a at the right PFO/P(VDF-TrFE) interface. The charge concentration at the interface is 2 × 10^19^ cm^−3^, it is ∼10^18^ cm^−3^ at a distance of 2.5 nm from the interface, and it drops to 10^15^ cm^−3^ in the centre of the PFO pillar. It is worth noting that in the centre of the PFO pillar the large energy barrier at the injecting contact depletes the semiconductor over the whole diode thickness. The charge carrier density in the accumulation layer is four orders of magnitude higher than that in the centre of the pillar. The origin of the accumulation is the lateral *x-*component of the polarization vector in the P(VDF-TrFE) slab.

### The role of the *x*-polarization

Based on the diode geometry, it is expected that the polarization is oriented along the *y*-direction. Hence, the formation of the *x-*polarization component is counterintuitive and it can be explained as follows. The large stray electric field between the polarization charges of the ferroelectric polymer and the compensating image charges in the electrode lowers the barrier, and enables the efficient charge injection at the bottom corner of the semiconductor–ferroelectric interface. The injected carriers set the local potential into the semiconductor equal to the bottom contact potential (Fig. 2c, *x*=265 nm), the electric field lines bend and a lateral *x-*component of the electric field (*F*_*x*_) rises. Consequently, the electric field lines are no longer parallel to the interface. When *F*_*x*_ is larger than the coercive field, a *P*_*x*_ polarization component is created and the displacement (*D*_*x*_) is enhanced. *D*_*x*_ is compensated by charge carriers, which further accumulate at the PFO/P(VDF-TrFE) interface. An accumulated channel along the whole interface from the bottom to the top contact is formed. The hole density evolution as a function of the applied voltage is discussed in Supplementary Note 6.

Figure 2d shows the *x-*polarization distribution in the P(VDF-TrFE) domains. The *x*-polarization is about 0.3 μC cm^−2^. Although the *x*-polarization is one order of magnitude lower than the *y*-polarization, it is high enough to maintain the charge accumulation. When the applied voltage is reduced, the *x-*polarization is retained by the ferroelectric polymer and, hence, the accumulated channel at the interface is preserved. This is confirmed by Supplementary Fig. 10, where the hole density at the right PFO/P(VDF-TrFE) interface with *V*=5 V is shown. Also in this case, a channel is accumulated. The *x*-polarization is perfectly symmetric around the centre of the PFO pillar, meaning that the negative polarization vectors at the left interface are mirrored by the positive polarization vectors at the right interface (further details are reported in Supplementary Note 7). The *x*-polarization is maximum (minimum) at right (left) side of the injecting bottom contact because of the stray field. The reverse situation holds at the collecting top contact because the stray field points in the opposite direction. Therefore, close to the extracting contact the charge carriers are pushed away from the interface. This results in a pinch-off of the accumulated channel.

### Scaling of the diode thickness

As a confirmation, Fig. 2e shows the charge carrier density (*p*) along the PFO/P(VDF-TrFE) interface between the two electrodes. The injecting bottom contact is located at *y*=265 nm, while the extracting top contact is located at *y*=0 nm. The injecting contact shows a 3 nm depletion region because of the width of the energy barrier at the contact, lowered by the stray field. The hole density is almost constant along the PFO/P(VDF-TrFE) interface. This situation is completely different from a space charge-limited diode, where the charge concentration decreases with the square root of the distance from the injecting contact (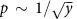)^30^ and the resulting current is described by the Mott–Gurney law^47^, which reads 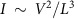, where *L* is the layer thickness. At the collecting top contact the charge concentration drops down to 10^17^ cm^−3^. The drop of the hole density is due to the stray field at the top contact that points in the opposite direction with respect to the injecting bottom contact (Fig. 2d). The hole density profile resembles that of a field-effect transistor operating in the saturation regime, where the drain current is proportional to 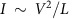, *V=V*_G_*−V*_T_, where *V*_G_ is the gate voltage and *V*_T_ is the threshold voltage^48^. In the memory diode the bottom contact is the source, the top contact is the drain and the gate voltage is equivalent to the drain voltage. This gives by definition a current proportional to 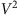. Although this is the same mathematical *I–V* relation as given by the Mott–Gurney law, the underlying physics is completely different. This can be further demonstrated by scaling the thickness *L* of the fabricated memory diodes (details in Supplementary Note 8). Figure 2f shows the measured on-current as a function of 1/*L* at *V*=10 V. The current linearly depends on the inverse of the thickness, and thus 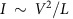 as in field-effect transistors operating at pinch-off.

### Charge injection mechanism

**Figure 3:**
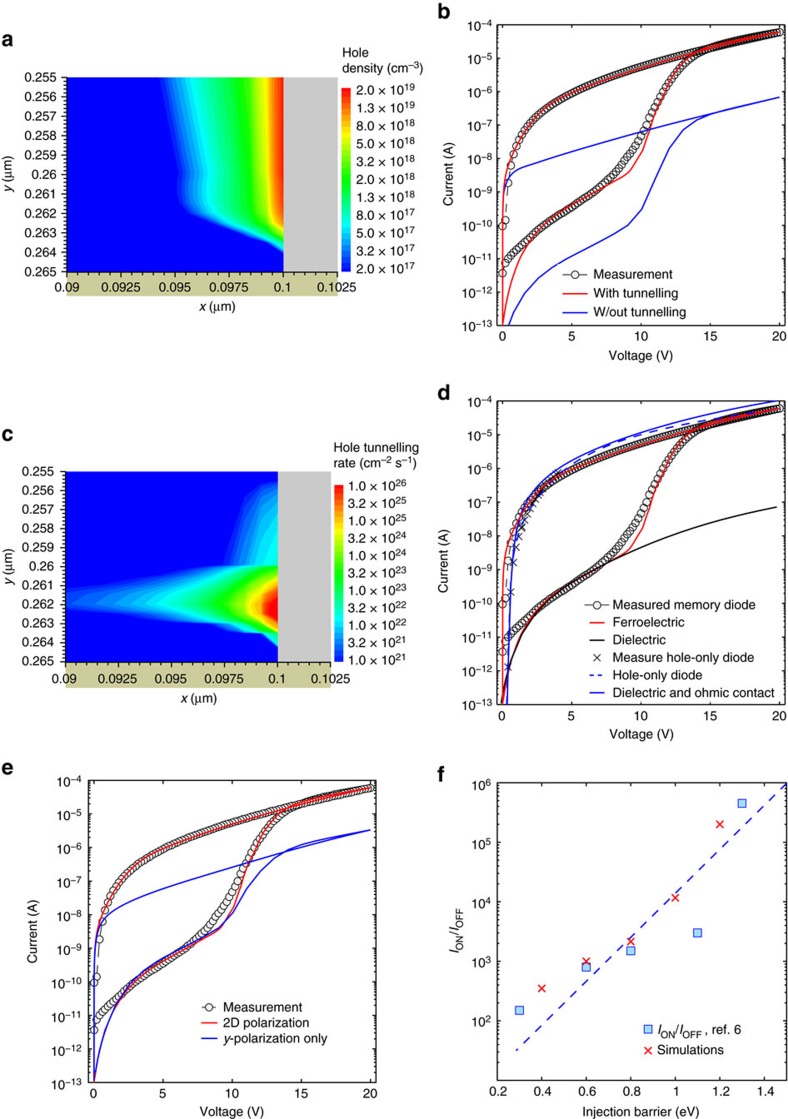
Injection and polarization in organic ferroelectric memory diodes. (**a**) Hole density distribution within the PFO at the interface-contact corner, *V*=20 V. (**b**) Measured (symbols) and simulated (lines) *I–V* characteristics at ambient temperature. The blue line is calculated by switching off the tunnelling. (**c**) Tunnelling rate within the PFO pillar at the interface-contact corner, *V*=20 V. (**d**) *I–V* characteristics calculated by varying the contact barrier and the ferroelectric properties. (**e**) *I–V* characteristics calculated accounting for the 2D polarization (*P*_*x*_+*P*_*y*_, red line) and only *y*-component *P*_*y*_ (blue line). (**f**) On/off current ratio as a function of injection barrier. Symbols correspond to experimental data taken from ref. 6, while the red crosses are the result of the simulations, and are obtained at *F*_*y*_=2 × 10^5^ V cm^−1^. The dashed line is the least-square approximation of the data taken from ref. 6. The slope is 0.25 eV dec^−1^.

Figure 3a shows the charge carrier concentration in the PFO pillar close to the P(VDF-TrFE) domain and the injecting electrode. The depletion width due to the contact barrier is less than 3 nm, which is compatible with tunnelling charge injection. To elucidate the contribution of the tunnelling on the charge injection current, Fig. 3b shows the *I–V* characteristics of a ferroelectric memory diode calculated with and without tunnelling injection. The model without tunnelling (blue line) does not explain the measured current, whereas the model including tunnelling charge injection (red line) perfectly describes the experimental data in both on- and off-states. A detailed analysis is shown in Supplementary Note 9. It is surprising that the thermionic emission can be disregarded with respect to the tunnelling. Figure 3c shows the hole tunnelling rate at the injecting contact. The tunnelling rate is as high as 10^26^ cm^−2^ s^−1^, efficiently providing all the charges transported in the accumulated channel. The width of the energy barrier is only 2.5 nm. The ferroelectric polymer is fully polarized and the tunnelling is due to the stray field (high local electric field) and to the accumulated channel (small depletion width). The metal–semiconductor Schottky contact behaves like an ideal Ohmic contact.

## Discussion

The ferroelectric polarization not only modulates the injection barrier but also yields a very high on-state current. In Fig. 3d we show the calculated *I–V* characteristics by varying both the injection barrier and the polarization. First, the ferroelectric polymer is replaced with a linear dielectric by turning off the ferroelectric polarization. The current (black curve) is contact limited. Next, we set the energy barrier to zero (Φ_B_=0 eV, blue full line) to obtain an ideal Ohmic contact. The resulting bulk-limited current is comparable to the current of a hole-only diode calculated by simply removing the P(VDF-TrFE) slab (dashed blue line). The hole-only diode current is slightly lower since the dielectric causes a weak charge accumulation in the PFO pillar. By restoring the original energy barrier at the contact (Φ_B_=1.3 eV) and switching on the ferroelectric polarization, the red curve is obtained. It perfectly reproduces the experimental data and surprisingly the memory diode current is comparable with that of the corresponding hole-only diode (viz. with equivalent semiconductor area as detailed in Supplementary Note 10). This can be explained as follows. In disordered organic semiconductors the mobility increases with the charge carrier density^28^,^49^. The increase of mobility combined with the strong hole accumulation compensates the reduction in the injection and transport area with respect to the corresponding bulk hole-only diode. This interpretation is confirmed by the weak temperature dependence of the on-state current. Figure 1d shows that on decreasing the temperature by 50 K the maximum on-current decreases only by a factor of 3 and the on-current at 3 V decreases by only one order of magnitude, which is reflected in low activation energies equal to 60 and 190 meV, respectively. The activation energy of hole-only diodes is typically much larger, between 200 and 600 meV^50^.

The lateral *x-*polarization is crucial to obtain such high on-state current. Figure 3e shows the impact of the *x-*component of the polarization vector. The blue curve is calculated by considering only the *y*-component of the polarization vector (**P**=*P*_*y*_). The current is more than one order of magnitude lower than that obtained with the 2D polarization vector (**P***=P*_*x*_*+P*_*y*_). The displacement given by the *x-*polarization is compensated by charges at the interface, which results in an accumulation layer. This already occurs in a linear dielectric where the displacement linearly depends on the electric field. However, in the ferroelectric domain the displacement is a nonlinear function of the electric field leading to an enhanced accumulation layer of only 2.5 nm (Fig. 2b). The shape of the *I–V* characteristics is mainly defined by the linear response of the ferroelectric polymer to the electric field, while the magnitude of the on-current depends on the *x*-polarization. This is readily confirmed by Fig. 3e. The on-current calculated accounting for only the *P*_*y*_ polarization has the same shape of that obtained accounting for both *P*_*x*_ and *P*_*y*_ polarization.

We calculate the on/off current ratio as a function of the injection barrier. The on/off current ratio is calculated at 5.3 V, which corresponds to a vertical electric field *F*_*Y*_=2 × 10^5^ V cm^−1^ to compare directly our results with ref. 6. The on/off current ratio is shown on a semilogarithmic scale as a function of barrier in Fig. 3f. A linear relation is obtained with a slope of 0.27 eV dec^−1^, which perfectly agrees with the experimentally extracted value of 0.25 eV dec^−1^ (ref. 6). We note that the much lower slope of 0.067 eV dec^−1^ reported previously^8^ is due to the underestimated injection-limited current since only thermionic emission was taken into account. This again confirms that tunnelling is the dominant injection mechanism.

**Figure 4:**
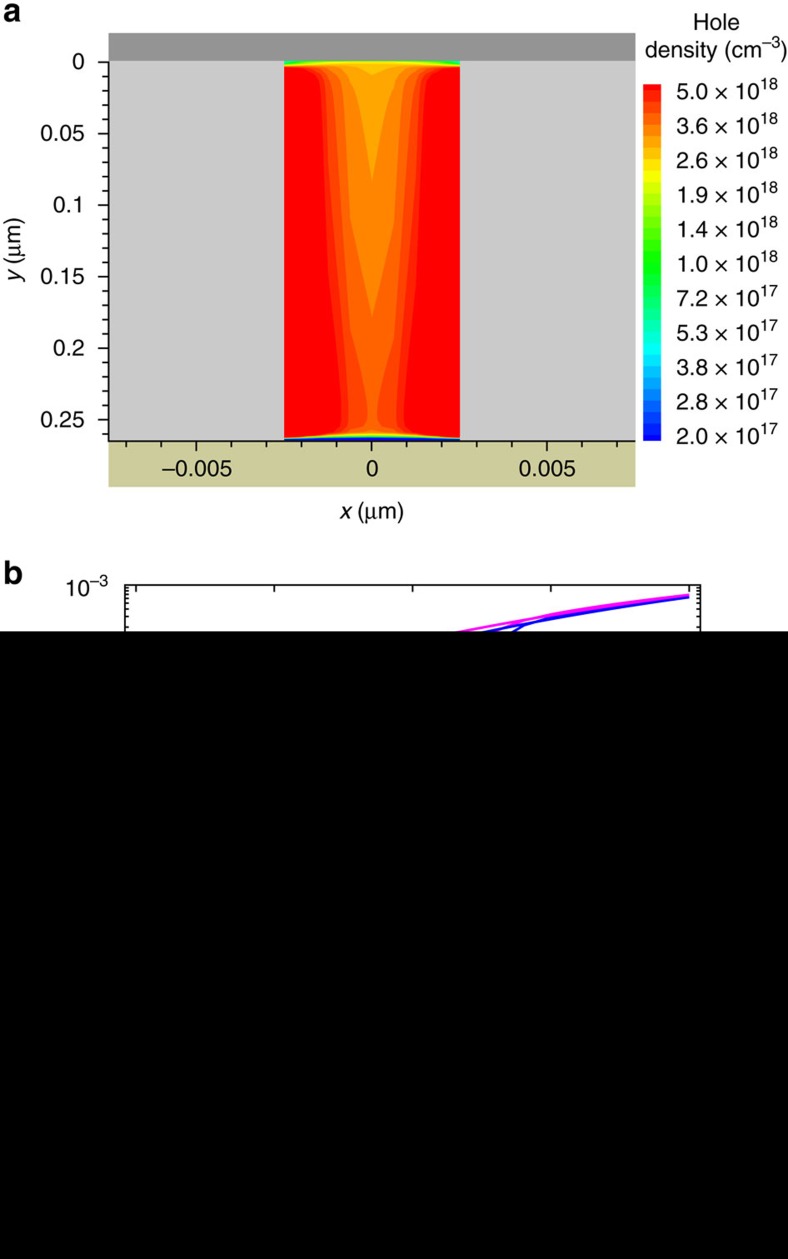
Scaling of organic ferroelectric memory diodes. (**a**) Hole concentration in the PFO pillar with lateral dimension *W*=*W*_PFO_+*W*_P(VDF-TrFE)_ , where *W*_PFO_=5 nm and *W*_P(VDF-TrFE)_=200 nm, biased at 5 V. (**b**) Comparison between the calculated *I–V* characteristics as a function of the P(VDF-TrFE) lateral dimension (*W*_P(VDF-TrFE)_) with *W*_PFO_=5 nm.

The strong confinement of both charge injection and charge transport shows that ferroelectric memory diodes are interface devices (Supplementary Note 11). We note that in previous reports current spreading within the semiconductor was calculated and, by considering only the dimension of the semiconductor slab, a minimum feature size of 50 nm was estimated^8^. Here we demonstrate that current spreading does not occur when the 2D polarization is taken into account. We found that both the charge injection and charge transport takes place in only 2.5-nm-thick channels. This enables the ultimate downscaling of the semiconductor lateral feature size down to 5 nm. Figure 4a shows the charge carrier density in a PFO pillar of only 5 nm surrounded by slabs of P(VDF-TrFE) of 200 nm. Although the applied bias is only 5 V, which corresponds to the operating voltage of the diode, two accumulated channels occur and extend over the whole semiconductor thickness.

A comprehensive evaluation of the minimum lateral dimensions (*W*) of a memory diode requires to consider the lateral dimensions of both the semiconductor (*W*_PFO_) and the ferroelectric polymer (*W*_P(VDF-TrFE)_), i.e., *W*=*W*_PFO_+*W*_P(VDF-TrFE)_. Therefore, we systematically varied *W*_P(VDF-TrFE)_, while keeping *W*_PFO_=5 nm constant; the device area is kept constant for the different cases, too. The resulting calculated *I–V* characteristics are presented in Fig. 4b. The off-state current is similar in all cases. If *W*_P(VDF-TrFE)_ is reduced from 200 nm down to 5 nm, the on-state current decreases. This is counterintuitive, as the decrease of the ferroelectric domain size results in an increase of the overall semiconductor area of the memory diode. Hence, a current increase is expected. However, the downscaling of *W*_P(VDF-TrFE)_ leads to a strong decrease of the *x*-polarization component (details are shown in Supplementary Note 12) and, therefore, the charge accumulation is reduced. Although the charge carrier density in the channel decreases with decreasing *W*_P(VDF-TrFE)_, Fig. 4b shows that, even for the smallest feature size *W*_P(VDF-TrFE)_=5 nm, a bistable diode is obtained with a current modulation of more than seven decades. This results in a maximum theoretical array density of about 10^12^ bit cm^−2^. Further discussions on device scaling and potential cross-talk between aggressively scaled devices are presented in the Supplementary Note 13.

In conclusion, we analyse the device physics of ferroelectric memory diodes based on a phase-separated blend of P(VDF-TrFE) and PFO. We demonstrate that to understand the operational mechanism it is crucial to take into account the 2D ferroelectric polarization explicitly. The full *I–V* characteristics are quantitatively described as a function of both bias and temperature. The key ingredient is the lateral polarization leading to the formation of a strongly accumulated hole density along the whole interface between the semiconducting and the ferroelectric polymer. Consequently, when the memory diode is operated in the on-state the current transport is not space charge limited but resembles the channel current in a field-effect transistor operated in saturation at pinch-off. This explains the 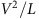 dependence of the current, the low activation energy and the large current density in the on-state. The current modulation as a function of the energy barrier is quantitatively explained. The stray field modulates the barrier width but, surprisingly, thermionic emission can be disregarded. We discover that in ferroelectric memory diodes, where large contact barriers are required for large on/off current ratio, charge injection is dominated by tunnelling. Since there is no current spreading into the semiconductor, the feature size can ultimately be scaled down to 10 nm, resulting in an ultimate theoretical array density of 10^12^ bit cm^−2^. The proposed model is general and can be applied for any combination of metal electrodes, semiconducting and ferroelectric polymers. The model leads to design rules for the implementation of ultrahigh-density organic non-volatile memories in several application fields including flexible displays, sensors, imperceptible and wearable electronics, biological and medical devices.

## Methods

### Device fabrication

The ferroelectric copolymer P(VDF-TrFE) with 65 mol% VDF and 35 mol% TrFE was purchased from Solvay. The number- and weight-average molecular weight, *M*_n_ and *M*_w_, were measured with gel permeation chromatography versus polystyrene standards and amounted to 147 and 296 kg mol^−1^, respectively. The polydispersity amounted to 2.01. PFO, purchased from TNO/Holst Centre, has *M*_w_=1,431 kg mol^−1^ and a polydispersity of 1.2. The PEDOT:PSS (Clevios P VP Al 4083) was purchased from Heraeus.

P(VDF-TrFE) and PFO were mixed in a ratio 9:1 by weight and then dissolved in cyclohexanone (45 mg of the polymer mixture in 1 ml cyclohexanone) at 80 °C under vigorous stirring. Before use, the solution was filtered (Teflon filter with pore size of 1 μm). Fifty-nm-thick Au lines were thermally evaporated on cleaned glass slides (Schott Borofloat) through a shadow mask. Two-nm-thick Cr is deposited before Au as it serves as adhesion layer. Afterwards, the samples were treated with UV/ozone, before the films were deposited via wire-bar coating using K202 control coater (RK PrintCoat Instruments, UK). The plate of the wire-bar coater was heated to 80 °C. Subsequently, the samples were annealed for 2 h at 140 °C in a vacuum oven to enhance the crystallinity of the ferroelectric P(VDF-TrFE). Film thickness was measured with a DEKTAK surface profilometer and amounted to *L*=265 nm.

Devices were fabricated in standard ambient conditions. To improve the yield, PEDOT:PSS was deposited by spin coating and used as top electrode^34^. The PEDOT:PSS top electrode was patterned by reactive ion etching and 50 nm Au lines, which serves as self-aligned mask, and were evaporated through a shadow mask. Therefore, the sample was exposed to oxygen plasma (plasma technology) for 3 min at a pressure of 0.1 mbar. Each cross-point of the resulting crossbar array has an area of ∼0.16 mm^2^, which is the device area of a discrete memory diode.

### Measurement set-up

AFM was performed in tapping mode using a Nanoscope Dimension 3100 (Bruker). Silicon tips with Al backside coating were used with a force constant of 26 N m^−1^ and a resonant frequency of ∼270 kHz. The electrical characteristics were acquired in vacuum at 10^−6^ mbar. The samples were fixed on a metal plate, which is connected to a cryostat (cooling with liquid N_2_) and to a temperature control unit (Oxford Intelligent Temperature Controller ITC 4). To ensure thermal contact, a layer of heat-conducting paste was applied between the plate and the substrate. Current–voltage characteristics were measured using a 4155B Semiconductor Parameter Analyser. The measurements were carried out only after the set temperature was stable for more than 10 min.

### Data availability

The data that support the findings of this study are available from the corresponding author on reasonable request.

## Additional information

**How to cite this article:** Ghittorelli, M. *et al*. Quantum tunnelling and charge accumulation in organic ferroelectric memory diodes. *Nat. Commun.* **8,** 15841 doi: 10.1038/ncomms15841 (2017).

**Publisher's note:** Springer Nature remains neutral with regard to jurisdictional claims in published maps and institutional affiliations.

## Supplementary Material

Supplementary Information
