# Supplementary material for: Quantum tunnelling and charge accumulation in organic ferroelectric memory diodes
Source: Nat Commun. 2017 Jun 12;8:15741. doi: 10.1038/ncomms15841 (PMC5477493; doi:10.1038/ncomms15841)
Supplement: Supplementary Information [file ncomms15841-s1.pdf]

Type of file: pdf
Size of file: 0 KB
Title of file for HTML: Supplementary Information
Description: Supplementary Figures, Supplementary Tables, Supplementary Notes and Supplementary References

Supplementary Note 1 | Numerical model and 2D simulations framework

The coupled Poisson, drift–diffusion, and current continuity equations are solved together on a two-dimensional (2D) grid. The Poisson equation includes the polarization, **P**, of a ferroelectric material and reads:

$$\nabla \cdot (\varepsilon_0 \varepsilon_\mathrm{r} \mathbf{F} + \mathbf{P}) = \rho \tag{1}$$

where ε_0 and ε_r are the vacuum permittivity and the relative permittivity, respectively. **F** is the electric field vector and **P** is the polarization vector.

The charge density ρ reads:

$$\rho = q(p - n + N_\mathrm{D}) \tag{2}$$

where q is the elementary charge, p and n are the hole and electron density, respectively, and N_D accounts for unintentional doping. The polarization **P** of the ferroelectric material nonlinearly depends on the electric field **F**.[1, 2] The polarization vector **P** is split into its components along the three main axes of the mesh coordinate system, and each component reads:[3, 4]

$$P_u = cP_\mathrm{s} \times \tanh\left[\frac{1}{2F_\mathrm{c}} \log\left(\frac{P_\mathrm{s}+P_\mathrm{r}}{P_\mathrm{s}-P_\mathrm{r}}\right) \times (F_u \pm F_\mathrm{c})\right] + P_\mathrm{off} \tag{3}$$

where $u \in (x, y, z)$, P_u is the polarization in the u-direction, F_u is the electric field in the u-direction, P_s, P_r, F_c are the saturation polarization, the remanent polarization and the coercive field, respectively. It is worth to note that P_s, P_r, F_c are homogeneous along the *x*, *y* and *z* direction, while *c* and P_off result from the polarization history of the material and enable to describe inner loops.[3, 4] In particular $c < 1$ accounts for the reduced magnitude of the inner loops, while P_off accounts for the polarization offset of the inner loops. It is worth noting that the plus sign applies to decreasing electric field and the minus sign applies to increasing electric field. We assume the separation between the polarization charges and the metal contact equal to the average inter-site distance, and in our case d_a = 1.5 nm.

The drift-diffusion and the current continuity equations, respectively, read

$$J_\mathrm{p} = \Gamma_\mathrm{p} \nabla \varphi_\mathrm{p} \tag{4}$$

$$J_\mathrm{n} = \Gamma_\mathrm{n} \nabla \varphi_\mathrm{n} \tag{5}$$

$$\frac{\partial p}{\partial t} = -\frac{1}{q} \nabla \cdot J_\mathrm{p} \tag{6}$$

$$\frac{\partial n}{\partial t} = \frac{1}{q} \nabla \cdot J_\mathrm{n} \tag{7}$$

where J_n and J_p are the electron- and hole-current densities, respectively, φ_n and φ_p are the pseudo-Fermi potentials of electrons and holes, respectively, and Γ_n and Γ_p are the electron- and hole-conductivity functional, respectively. Γ_n and Γ_p are calculated by means of the VRH model and accounts for the energy disorder. In the specific case of our ferroelectric memory diodes the electron transport can be disregarded because the electron contact barrier is significantly larger than the hole barrier, and therefore we set Γ_n = 0. The hole conductivity functional Γ_p accounts for the VRH model and energy disorder, and it is calculated as follows.

We numerically calculate the conductivity of the organic semiconductor basing on the general framework of the percolation theory and assuming a Gaussian density of states (DOS). This extends the framework used by Vissenberg and Matters[5] to the case of a Gaussian DOS, eventually providing a numerical solution. More in detail, the system (viz. disordered organic semiconductor) is modelled as a random resistor network.[6] The microscopic conductance between two different energy sites *i* (donor site) and *j* (acceptor site) is given by

$$G_{ij} = \mathrm{f}(E_i, E_\mathrm{F})\left[1 - \mathrm{f}\left(E_j, E_\mathrm{F}\right)\right] \nu_{ij} \tag{8}$$

where E_F is the Fermi energy, E_i and E_j are the energy levels of the donor and acceptor site, respectively, and $f(E,E_\mathrm{F}) = \{1+\exp[(E-E_\mathrm{F})/(k_\mathrm{B}T)]\}^{-1}$ is the Fermi-Dirac occupation probability. ν_{ij} is the tunnelling rate between two sites, and we assumed the Miller-Abrahams hopping model[7] that reads

$$\nu_{ij} = \nu_0 \exp\left(-2\alpha r_{ij}\right) \cdot \exp\left[-\frac{E_j - E_i}{k_\mathrm{B}T} \cdot \Theta\left(E_j - E_i\right)\right] \tag{9}$$

where ν_0 is the attempt-to-escape frequency, r_{ij} is the distance between the donor and the acceptor site, α is the inverse of the localization radius, k_B is the Boltzmann constant, and T is the temperature. The Heaviside function $\Theta(x) = 1$ if $x > 0$ while $\Theta(x) = 0$ if $x < 0$. The microscopic conductance can be rewritten as

$$G_{ij} = G_0 \exp(-s_{ij}) \tag{10}$$

$$s_{ij} = 2\alpha r_{ij} + \frac{|E_i - E_\mathrm{F}| + |E_j - E_\mathrm{F}| + |E_i - E_j|}{2k_\mathrm{B}T} \tag{11}$$

where $G_0 = q^2\nu_0/(k_\mathrm{B}T)$,[8] and q is the elementary charge. By making use of the percolation theory, the conductance of an organic semiconductor can be obtained by considering all the microscopic conductances such that $G_{ij}>G_\mathrm{c}$, where $G_\mathrm{C} = G_0 \exp(-s_\mathrm{c})$ is the critical percolation conductance of the system and s_c is the critical exponent. The macroscopic conductivity Γ_p can therefore be calculated as

$$\Gamma_\mathrm{p} = \Gamma_{00} \exp(-s_\mathrm{c}) \tag{12}$$

where Γ_{00} is proportional to G_0/d_a.[8] The onset of percolation is determined by calculating the average number of bonds per sites

$$B_\mathrm{c} = \frac{N_\mathrm{b}(s_\mathrm{c}, E_\mathrm{F})}{N_\mathrm{s}(s_\mathrm{c}, E_\mathrm{F})} \tag{13}$$

where $B_\mathrm{c} \approx 2.8$,[6,8] and N_b is the density of bonds that satisfy the percolation criterion and it can be calculated as follows

$$N_\mathrm{b} = 4\pi \int_{\mathbb{R}^3} r_{ij}^2 g(E_i) g\left(E_j\right) \Theta\left(s_\mathrm{c} - s_{ij}\right) \mathrm{d}E_j \mathrm{d}E_i \mathrm{d}r_{ij} \tag{14}$$

and $g(E)$ is the Gaussian DOS defined as

$$g(E) = \frac{N_\mathrm{t}}{\sqrt{2\pi}\sigma} \exp\left[-\frac{(E - E_\mathrm{HOMO})^2}{2\sigma^2}\right] \tag{15}$$

where $N_\mathrm{t} = 1/d_\mathrm{a}{}^3$ is the total density of states, σ is the DOS energy width and E_HOMO is the highest occupied molecular orbital (HOMO) energy level. N_s is the density of sites that satisfy the percolation criterion, and it reads

$$N_\mathrm{s} = \int_{\mathbb{R}} g(E)\Theta(s_\mathrm{c}k_\mathrm{B}T - |E - E_\mathrm{F}|)\mathrm{d}E \tag{16}$$

By numerically solving $N_\mathrm{b}(s_\mathrm{c},E_\mathrm{F})/N_\mathrm{s}(s_\mathrm{c},E_\mathrm{F}) - B_\mathrm{c} = 0$ (viz. Eq. 13 with Eqs. 14-16), the macroscopic conductivity Γ_p as a function of Fermi energy E_F, temperature T, average inter-site distance d_a, energy width σ, and inverse of the localization radius α is numerically calculated. In the case of a Gaussian DOS it can be approximated as[8,9]

$$\Gamma_\mathrm{p} = \Gamma_0 \exp\left(\frac{E_\mathrm{F}}{k_\mathrm{B}T}\right) \tag{17}$$

where Γ_0 depends on T, d_a, v_0, σ, and α. Finally, the hole mobility functional can be calculated as follows

$$\mu_\mathrm{p} = \frac{\Gamma_\mathrm{p}}{qp} = \frac{\Gamma_0}{qp}\exp\left(\frac{E_\mathrm{F}}{k_\mathrm{B}T}\right) \tag{18}$$

where p is the hole concentration calculated as

$$p = \int_{\mathbb{R}} g(E)[1 - f(E, E_\mathrm{F})]\mathrm{dE} \tag{19}$$

We found that the mobility functional (Eq. 18) is in agreement with the model based on the Master equation presented by Coehoorn et al. (Eq. 30).[8] The latter, in turn, is equivalent to the model of Pasveer et al.,[10] when the electric field enhancement of the mobility can be disregarded (i.e. zero-field approximation). It is worth noting that, according with Coehoorn et al.,[8] Γ_0 after straightforward manipulation reads

$$\Gamma_0 = \frac{q^2 v_0 N_\mathrm{t}^{1/3}}{k_\mathrm{B}T}\Phi \exp\left(-\widehat{p_0} - \frac{a\,\sigma}{k_\mathrm{B}T} + \frac{d}{\widehat{p_0}}\frac{\sigma^2}{k_\mathrm{B}^2T^2}\right) \tag{20}$$

where $\widehat{p_0}$, a and d are fitting parameters, which depend on N_t/α^3. In our work we numerically calculated Γ_0 as a function of temperature, at T = [250, 260, 270, 280, 290, 300] K and, in the case of a Gaussian DOS, this is equivalent of using Eq. (20) with the fitting parameters derived in Ref. 8.

According to Holst et al.[11] the electric field enhancement factor of the mobility decreases by increasing the disorder and the zero-field approximation is valid when the energy disorder is large. In the case of our ferroelectric memory diodes σ = 0.16 eV, we estimated that the increase of the current density due to the electric field enhancement of the mobility is about 15%, and therefore the zero-field approximation can be used.

The charge flow at the injecting and collecting contacts is calculated accounting for charge drift and diffusion, energy disorder, image force barrier lowering, thermionic emission and the tunnelling.

According to Holst et al.,[11] as long as the device thickness is larger than 102 nm, filamentary current transport can be disregarded and a 1D continuum drift-diffusion model can be used. This condition is fulfilled in our ferroelectric memory diodes (L = 265 nm). In order to numerically solve the drift-diffusion

equation, the VRH model with a Gaussian DOS is accounted for by means of the conductivity functional (Eq. 17) and the charge carrier density at the metal-semiconductor contact (boundary condition) is calculated as

$$p_0 = N_{\mathrm{t}} \exp\left(-\frac{\Phi_{\mathrm{B}}}{k_{\mathrm{B}}T}\right) \tag{21}$$

where $\Phi_B = \Phi_{B0} - \Delta\Phi_D - \Delta\Phi_{BL}$ is the effective barrier, which accounts for the energy disorder ($\Delta\Phi_D$) and the image force barrier lowering ($\Delta\Phi_{BL}$). The energy barrier $\Phi_{B0} = E_{HOMO} - \Phi_m$ is the contact energy barrier given by the energy misalignment between the HOMO level of the organic semiconductor and the electrode work function. According with [8, 12, 13] $\Delta\Phi_D$ can be calculated from the analytical approximation of Eq. 19, calculated at the contact where $E_F = \Phi_m$, and reads

$$\int_{-\infty}^{+\infty} \frac{g(E)}{1+\exp[(E - E_{\mathrm{HOMO}} + \Phi_{\mathrm{B0}})/k_{\mathrm{B}}T]}\, dE \approx N_{\mathrm{t}} \exp\left(-\frac{\Phi_{\mathrm{B0}}}{k_{\mathrm{B}}T}\right) \exp\left(\frac{1}{2}\hat{s}^2\right) \tag{22}$$

where $\hat{s} = \sigma/k_BT$ and therefore $\Delta\Phi_D = \sigma^2/2k_BT$.

The image force barrier lowering $\Delta\Phi_{BL}$ reads[14, 15]

$$\Delta\Phi_{\mathrm{BL}} = e\sqrt{\frac{eF_0}{4\pi\varepsilon_0\varepsilon_{\mathrm{r}}}} \tag{23}$$

where F_0 is the positive electric field at the contact, ε_0 is the vacuum permittivity and ε_r is the relative permittivity of the semiconductor, in our case $\varepsilon_{rPFO} = 3$ (Supplementary Table 1). By substituting the effective barrier Φ_B to the contact barrier Φ_{B0} in p_0, (Eq. 21) our model is equivalent to the 1D continuum model proposed by Holst et al.[11]

In our memory diodes, we assumed an energy barrier between the gold electrode and the PFO HOMO level Φ_{B0} = 1.3 eV,[16] from the measurements of hole-only diodes we found σ = 0.16 eV and this results in a $\Delta\Phi_D \approx 0.5$ eV. Therefore, the effective contact barrier is calculated as Φ_B = 0.8 eV - $\Delta\Phi_{BL}$ where $\Delta\Phi_{BL}$ is given by Eq. 23.

In addition, we included both thermionic emission and tunnelling as possible mechanisms for charge injection. Importantly, we want to stress that both the thermionic emission model and the tunnelling model take into account the disorder of the organic semiconductor.

The thermionic emission hole current density is calculated as

$$J_{\mathrm{th}} = \frac{A^*T^2}{N_{\mathrm{t}}}(p - p_0) \tag{24}$$

where $A^* = 4\pi q m^* k_B^2 / h^3$ is the effective Richardson constant, h is the Planck's constant, p and p_0 are calculated with Eqs. 19 and 21, respectively, and m^* is the hole effective mass. In organic semiconductors, the latter is typically in the range of 0.8 – 1.3 the electron rest mass $m_0 = 9.11\ 10^{-31}$ kg. For simplicity we assumed $m^* = m_0$.

The tunnelling current density is calculated as[17]

$$J_{\mathrm{tu}} = \int_{-\infty}^{\infty} \frac{A^{*}T}{k_{\mathrm{B}}} \chi_{\mathrm{p}}(r,E) \times \left[\log\left(1+\exp\left[\frac{E_{\mathrm{F}}(r)-E}{k_{\mathrm{B}}T}\right]\right) - \log\left(1+\exp\left[\frac{E_{\mathrm{F}}(0^{-})-E}{k_{\mathrm{B}}T}\right]\right)\right] \mathrm{d}E \qquad (25)$$

where $E_F(r)$ is the position dependent Fermi energy, r is the distance from the metal/semiconductor interface, E is the energy, and χ_p is the tunnelling probability based on the Wentzel–Kramers–Brillouin (WKB) approximation that reads[15]

$$\chi_{\mathrm{p}}(r,E) = \exp\left(-\frac{2}{\hbar}\int_{0}^{r}\sqrt{2m^{*}\ |E_{\mathrm{HOMO}}(r)-E|}\,\mathrm{d}r\right)\Theta[E_{\mathrm{HOMO}}(r)-E] \qquad (26)$$

where $\hbar = h/2\pi$. The simulations parameters are listed in Supplementary Table 1. The charge transport parameters of the semiconductor (Γ_0, d_a, σ) are obtained from the measurements of PFO hole-only diodes (Supplementary Figure 1), the parameters of the ferroelectric polymer ($\varepsilon_{rP(VDF\text{-}TrFE)}$, P_s, P_r) are obtained from the measurements of P(VDF-TrFE) capacitors (Supplementary Figure 2), the unintentional doping (N_D) is estimated from the off-current of ferroelectric memory diodes (Supplementary Figure 4), and the other material and physical parameters (Φ_{Au}, $\Phi_{PEDOT:PSS}$, E_{HOMO}, E_{LUMO}, ε_{rPFO}, m^*) are in agreement with previously reported values.[18,19,20-24] Finally, it is worth noting that the drift-diffusion, thermionic and tunnelling equations including energy disorder and image force barrier lowering are coupled (viz. solved together) with the Poisson equation in our two-dimensional device model.

Supplementary Table 1 | Simulation parameters.

Work function of gold	Φ_{Au} = 4.5 eV	Ref. 19
Work function of PEDOT:PSS	$\Phi_{PEDOT:PSS}$ = 5.0 eV	Ref. 22
Highest occupied molecular orbital (HOMO)	E_{HOMO} = 5.8 eV	Refs. 18, 21, 23
Lowest unoccupied molecular orbital (LUMO)	E_{LUMO} = 2.8 eV	Refs. 18, 21
Relative permittivity of PFO	ε_{rPFO} = 3	Ref. 24
Hole effective mass	m^* = 9.11 x 10^{-31} kg	Ref. 20
Attempt to escape frequency	v_0 = 10^{14} Hz	Ref. 25
VRH conductivity prefactor at T = 290 K	Γ_0 = 2 x 10^{-5} S cm^{-1}	Hole-only diodes
Intersite distance	d_a = 1.5 nm	Hole-only diodes
DOS energy width	σ = 0.16 eV	Hole-only diodes
Unintentional doping of PFO	N_D = 1 x 10^{17} cm^{-3}	Memory diodes off-current
Relative permittivity of P(VDF-TrFE)	$\varepsilon_{rP(VDF\text{-}TrFE)}$ = 14	P(VDF-TrFE) capacitors
Saturation polarization	P_s = 7.001 x 10^{-6} C cm^{-2}	P(VDF-TrFE) capacitors
Remanent polarization	P_r = 7 x 10^{-6} C cm^{-2}	P(VDF-TrFE) capacitors
Coercive field at 290 K	F_c = 5.3 x10^{5} V cm^{-1}	P(VDF-TrFE) capacitors

Supplementary Note 2 | PFO hole-only diodes

In order to extract the hole charge transport parameters of the PFO batch used, we fabricated hole-only diodes, where PFO is sandwiched between indium tin oxide (ITO) and MoO_x electrodes. MoO_x was chosen because it forms an Ohmic contact with PFO.[23] PFO dissolved in toluene was spincoated onto a pre-patterned ITO slide, covered with a 45 nm PEDOT:PSS (Clevios P VP 4083, Heraeus) film. The PFO film thickness amounted to 210 nm. As a top contact, 10 nm MoO_x capped with 100 nm Al was evaporated at a pressure of 10^{-6} mbar. The diode area amounted to 8 mm^2. The measured *I-V* characteristics of the hole-only diode at temperatures between 235 K and 295 K are presented in Supplementary Figure 1. The solid lines are fits to the data using the numerical framework presented in Supplementary Note 1. A good agreement is obtained. The extracted charge transport parameters are presented in Supplementary Table 1.

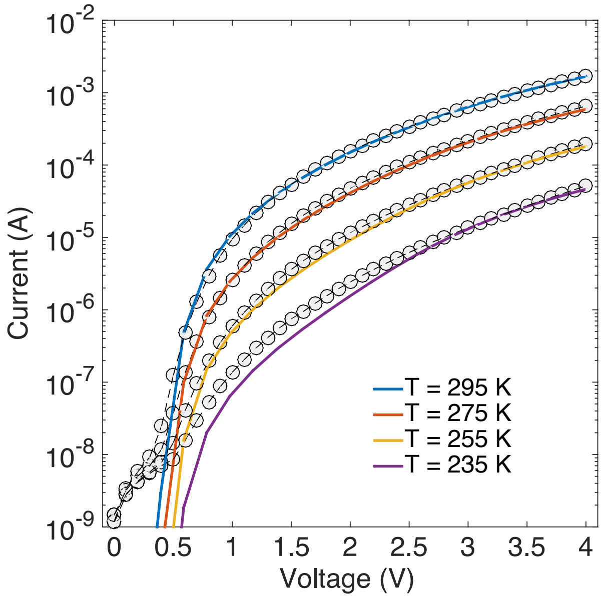

Supplementary Figure 1 | Temperature dependent *I-V* characteristics of a PFO hole-only diode. The current as a function of voltage is measured at several temperatures. Symbols represent the measurements, while the solid lines represent fits to the data. The PFO film thickness and the device area amounted to 210 nm and 8 mm^2, respectively.

Supplementary Note 3 | Extraction of ferroelectric parameters from measurements of thin film capacitors

Ferroelectric hysteresis loops were measured for P(VDF-TrFE) thin film capacitors using a Sawyer-Tower circuit at 1 kHz. Supplementary Figure 2 shows the displacement-electric field loops measured at temperatures between 210 K and 290 K. The saturated polarization is about equal to the remanent polarization and amounts to about 75 mC/m^2. The polarization is almost temperature independent. The coercive field is strongly temperature dependent and varies from 60 MV/m at ambient temperature to 150 MV/m at 210 K.

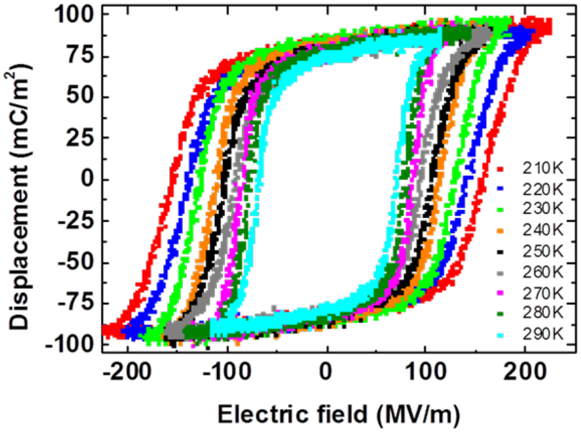

Supplementary Figure 2 | Temperature dependent displacement-electric field loops. Ferroelectric hysteresis loops measured on a P(VDF-TrFE) thin film capacitors using a Sawyer-Tower circuit at 1 kHz. The temperature was varied between 210 K and 290 K.

The memory diodes are operated in quasi steady-state. The values of the quasi static coercive field however cannot be directly obtained from the dynamically measured hysteresis loops as these loops are frequency dependent.[26-30] In order to obtain the quasi-static values, we iteratively calculate the hysteresis loop using the Kologorov-Avrami-Ishibashi (KAI) model.[31-34] The KAI model is based on the statistics of the domain nucleation and growth. The polarization transient during switching is derived as a compressed exponential function, which is expressed as:

$$\Delta P(t)/2P_r = 1 - \exp\left[-\left(\frac{t}{t_0}\right)^n\right] \tag{27}$$

where the switching parameters t_0 and n are extracted from polarization switching transients as input.[33] By means of the parameters extracted, we reconstruct the hysteresis loops. We take a triangular profile for the time-varying electric field. Then, at each time t, the electric field is known. The polarization at time $t + \Delta t$ follows from the polarization at time t and it reads:

$$P(t+\Delta t) = P(t) + \left[P(\infty) - P(t)\right]\cdot\left[1 - e^{-(t/t_0)^n}\right] \tag{28}$$

where $P(\infty)$ is the saturated polarization. Hence the polarization is numerically calculated as a function of time and converted into polarization as a function of electric field. From the reconstructed hysteresis loops at a frequency of 1 mHz, the quasi-static coercive field is extracted and plotted as a function of temperature in Supplementary Figure 3. For comparison, the experimental values of the coercive field measured at 1 kHz are included.

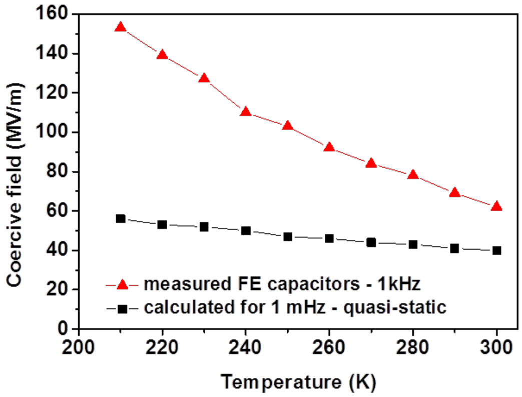

Supplementary Figure 3 | Values of the coercive field of P(VDF-TrFE) as a function of temperature. The black symbols represent values for the quasi-static coercive field, calculated at a frequency of 1 mHz. The red symbols represent the values for the coercive field measured using a Sawyer-Tower circuit at a frequency of 1 kHz.

Supplementary Note 4 | Estimation of the unintentional doping

The unintentional doping N_D was estimated by reproducing the memory diode off-current. More in detail, Supplementary Figure 4 shows the *I-V* characteristics of a typical ferroelectric memory diode. When it is measured for the first time (pristine diode) the off-current is significantly lower than that of the very same diode measured for the second time (dashed line). It is worth noting that between two subsequent measurements the diode is "erased" by applying a large negative voltage V = -20 V (not shown in Supplementary Figure 4).

We measured the diode many times and the current always overlapped with the second measurement (dashed line). We believe that charges are introduced into the device during the first measurement and these charges significantly increase the off-current. By means of 2D numerical simulations we estimated $N_D = 5 \times 10^{16}$ cm^{-3} in the case of the pristine device (1st measurement), and $N_D = 1 \times 10^{17}$ cm^{-3} in the case of the subsequent measurements. Similar values of unintentional doping have been recently measured in π-conjugated polymeric semiconductors.[35-38] We speculate that such a high unintentional doping level could be due to charged defects and/or adjacent cation-anion pairs resulting from the twisting of a planar conjugated polymer backbone.

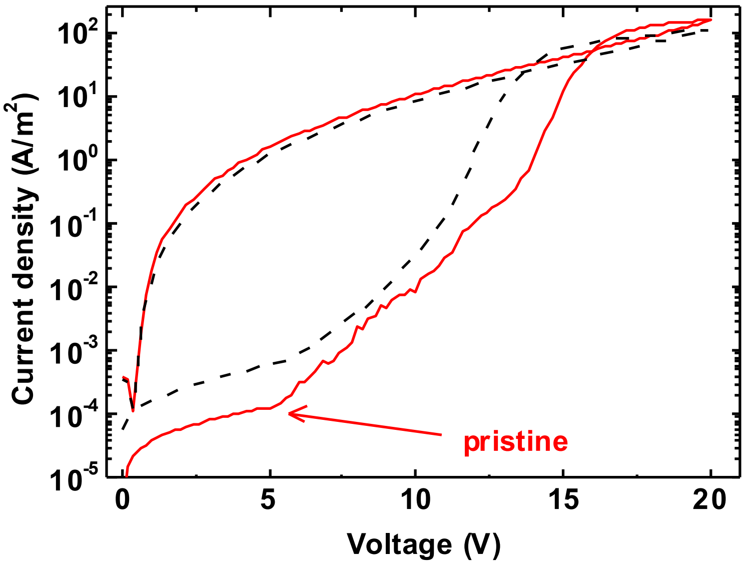

Supplementary Figure 4 | Measured *J-V* characteristics of a ferroelectric memory diode measured several times. Each time the memory diode is programmed (i.e. positive voltage sweep) and erased (i.e. negative voltage sweep).

Supplementary Note 5 | Morphological analysis

In order to determine the area factor for the numerical simulations we performed AFM measurements in tapping mode at 6 different positions on the phase-separated blend film. Supplementary Figure 5 shows the measured AFM micrographs, where bicontinuous PFO pillars of different diameter are embedded in a matrix of semicrystalline P(VDF-TrFE). For each micrograph, we extracted the overall circumference of the PFO domains, which corresponds to the total interface length within a 5x5 μm^2 area. According with the statistics obtained for the different micrographs (Supplementary Table 2), we used an average value of 16.8 µm, and the area factor is determined as:

$$area\ factor = \frac{1}{2} \cdot 16.8\ \mu\mathrm{m}\ \cdot \frac{1.6 \cdot 10^{-7}\mathrm{mm}^2}{5\ \mu\mathrm{m} \cdot 5\ \mu\mathrm{m}} = 5.4 \cdot 10^4\ \mu\mathrm{m},$$

where ½ accounts for the two interfaces in the numerical simulations. The total device area is 1.6 x 10^{-7} mm^2.

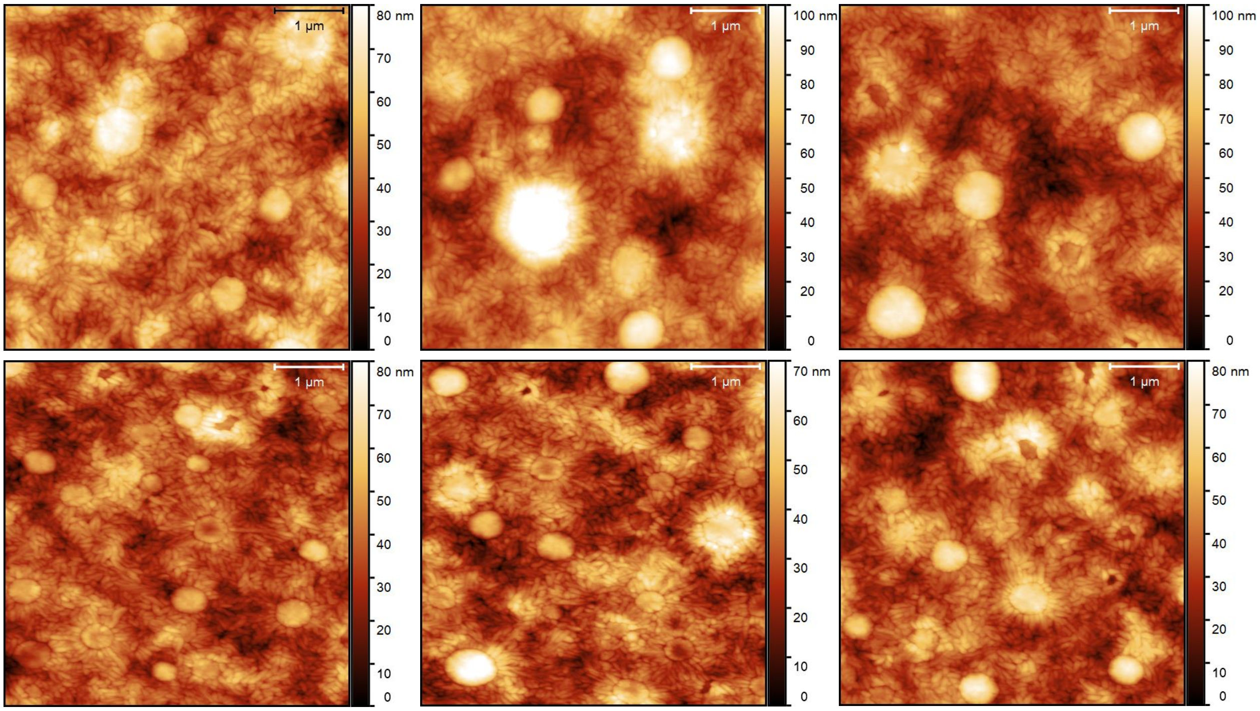

Supplementary Figure 5 | AFM micrographs of a phase separated blend film. AFM micrographs at six different positions on a typical P(VDF-TrFE)/PFO thin film. The PFO domain diameters were thoroughly analyzed. From the circumference of the domains, the effective P(VDF-TrFE)/PFO interface length was determined.

Supplementary Table 2 | Statistics for the different micrographs

Position	Sum circumference (µm)	Thickness (nm)	RMS
1	13.5	283	14.1
2	17.4	267	11.5
3	15.6	268	14.4
4	20.5	256	9.9
5	17.3	273	10.8
6	16.4	253	12.3
**Average**	**16.8**	**267**	**12.2**
**Standard deviation**	2.3 (13.8%)	11 (4%)	1.8 (15%)

To further analyse the bicontinuity of the semiconducting domains embedded in a P(VDF-TrFE) matrix, we performed AFM characterization of the phase separated P(VDF-TrFE)/PFO blends before and after selective dissolution of the PFO phase. Supplementary Figure 6a shows the AFM micrograph after selective dissolution of PFO, obtained by immersing the sample in toluene. The profile lines are shown in Supplementary Figure 6b. Profiles 1 and 2 are taken across a PFO-removed domain and clearly show that the PFO domains are continuous and the bottom of the substrate is reached even when the diode thickness is about 400 nm. We note that in the case some of the PFO columns are not continuous they do not contribute to the diode current, and hence, the total active area and interface length of the memory diode has to be calculated accordingly.

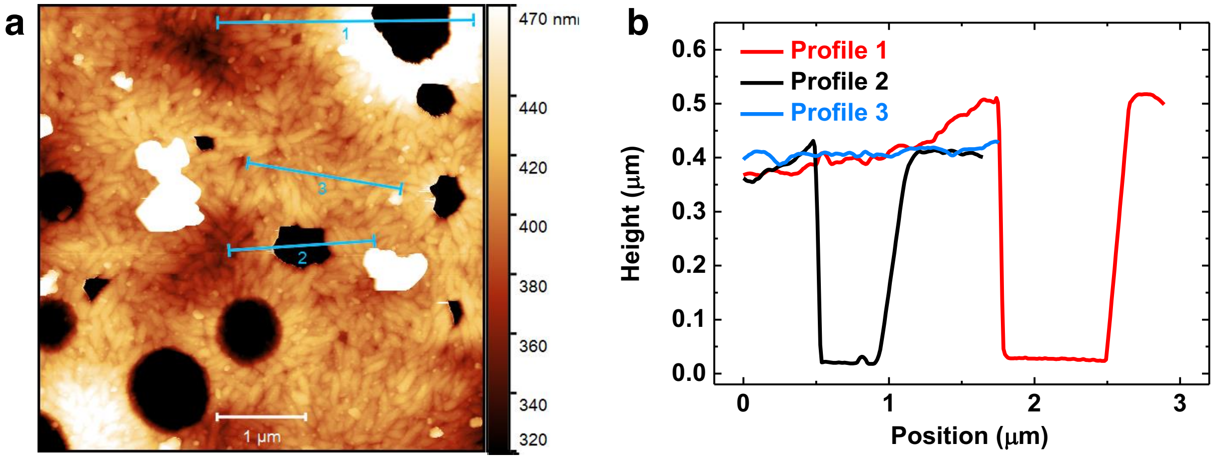

Supplementary Figure 6 | Continuity analysis of PFO columns. (**a**) AFM characterization and (**b**) profiles of the phase separated P(VDF-TrFE)/PFO blend after selective dissolution of the PFO.

Finally, we tackled the scaling limit by varying the composition of the blend solution. According with our previous work,[39] we decreased the amount of PFO in the P(VDF-TrFE):PFO blend solution and the SEM images for 90:10 and for 98:2 concentrations are presented in Supplementary Figure 7. In the case 90:10 the typical domain size is about 500 nm, in agreement with the AFM study in the Supplementary Figure 5. By decreasing the amount of semiconductor, viz. 98:2, the domain size can be significantly decreased, however the average domain size is still in the range of 100-200 nm. In order to further reduce the PFO domain size, a viable approach could be based on the combination of nano-imprint lithography (NIL)[40-42] with the backfilling procedure previously published by our group.[43,44] From this perspective, we hope that our theoretical and experimental work will trigger new technological studies on this topic, thus paving the way to aggressively scaled ferroelectric organic memory diodes.

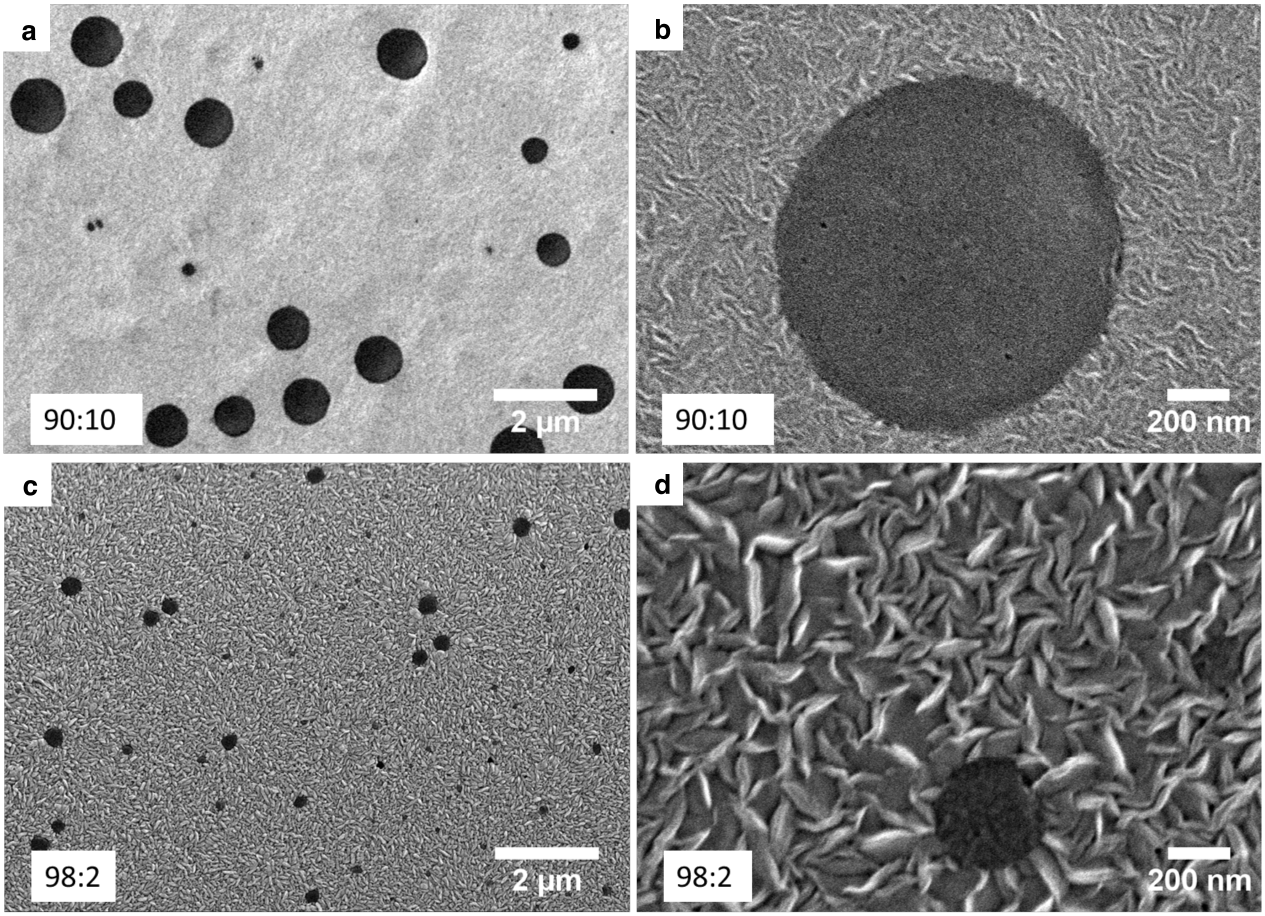

Supplementary Figure 7 | SEM analysis. SEM topography images (top view) of P(VDF-TrFE):PFO blends. (**a**) The concentration of the blend solution is 90:10. (**b**) Zoom of panel a. (**c**) The concentration of the blend solution is 98:2 and (**d**) Zoom of panel c.

Supplementary Note 6 | Evolution of the interface accumulation layer in a symmetric ferroelectric memory diode

Supplementary Figure 8 (full line) shows the calculated *I-V* characteristic of a symmetric device, where the bottom and top contacts have the same barrier Φ_{B0} = 1.3 eV. The applied voltage is swept from -20 V to 20V. Supplementary Figure 9 shows the hole distribution within the semiconductor as a function of the applied voltage. When the ferroelectric memory diode is biased at large negative voltage (V = -20 V) holes are injected at the top contact, and accumulated and transported at the PFO/P(VDF-TrFE) interface (Supplementary Figure 9a). The accumulation layer is sustained by the x-polarization. By increasing the voltage from -5 V to -1.25 V the length of the accumulation layer is progressively reduced (Supplementary Figures 9b-e). At V = 0 V, the accumulation layer is about 60 nm long and located near the top contact (Supplementary Figure 9f). When a small and positive voltage is applied, holes are weakly injected by the bottom contact but the stray field points to the opposite side with respect to the electric field. As a consequence, two small channels are still accumulated at the PFO/P(VDF-TrFE) interfaces close to the top contact (Supplementary Figure 9g). Further increasing the voltage, the charge accumulation near the top electrode progressively disappears (Supplementary Figure 9h-j). At the coercive voltage (here about 10 V), the ferroelectric polymer is polarized and the stray field at the PFO/PVDF-TrFE interface enables the efficient charge injection from the bottom contact. The hole concentration in the accumulation layer is about 10^{18} cm^{-3} at 12.25 V (Supplementary Figure 9k), 5 10^{18} cm^{-3} at 13.75 V (Supplementary Figure 9l), and exceeds 10^{19} cm^{-3} at 15 V (Supplementary Figure 9m). The latter is comparable with the charge concentration obtained at the maximum voltage V = 20 V (Supplementary Figure 9n).

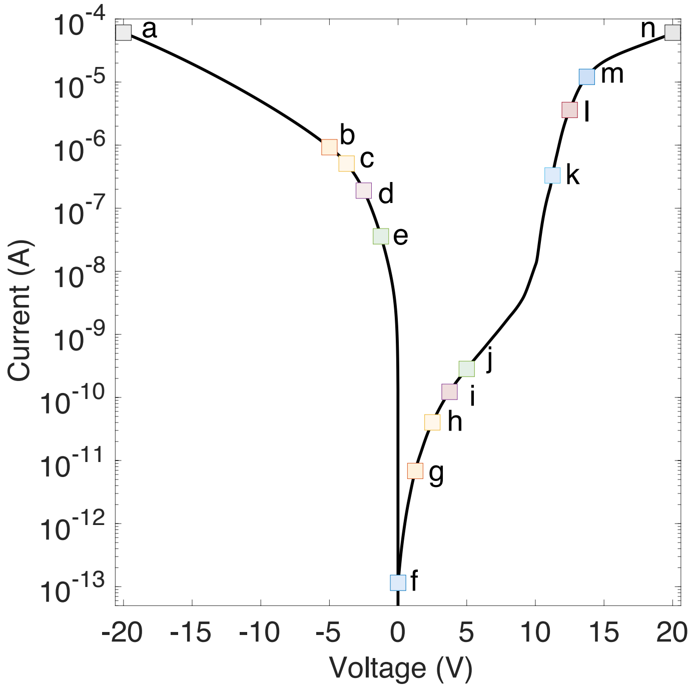

Supplementary Figure 8 | Current-voltage characteristic of a symmetric memory diode. Numerically calculated *I-V* characteristic of a memory diode with symmetric energy barriers at the contacts. The barrier height is Φ_{B0} = 1.3 eV.

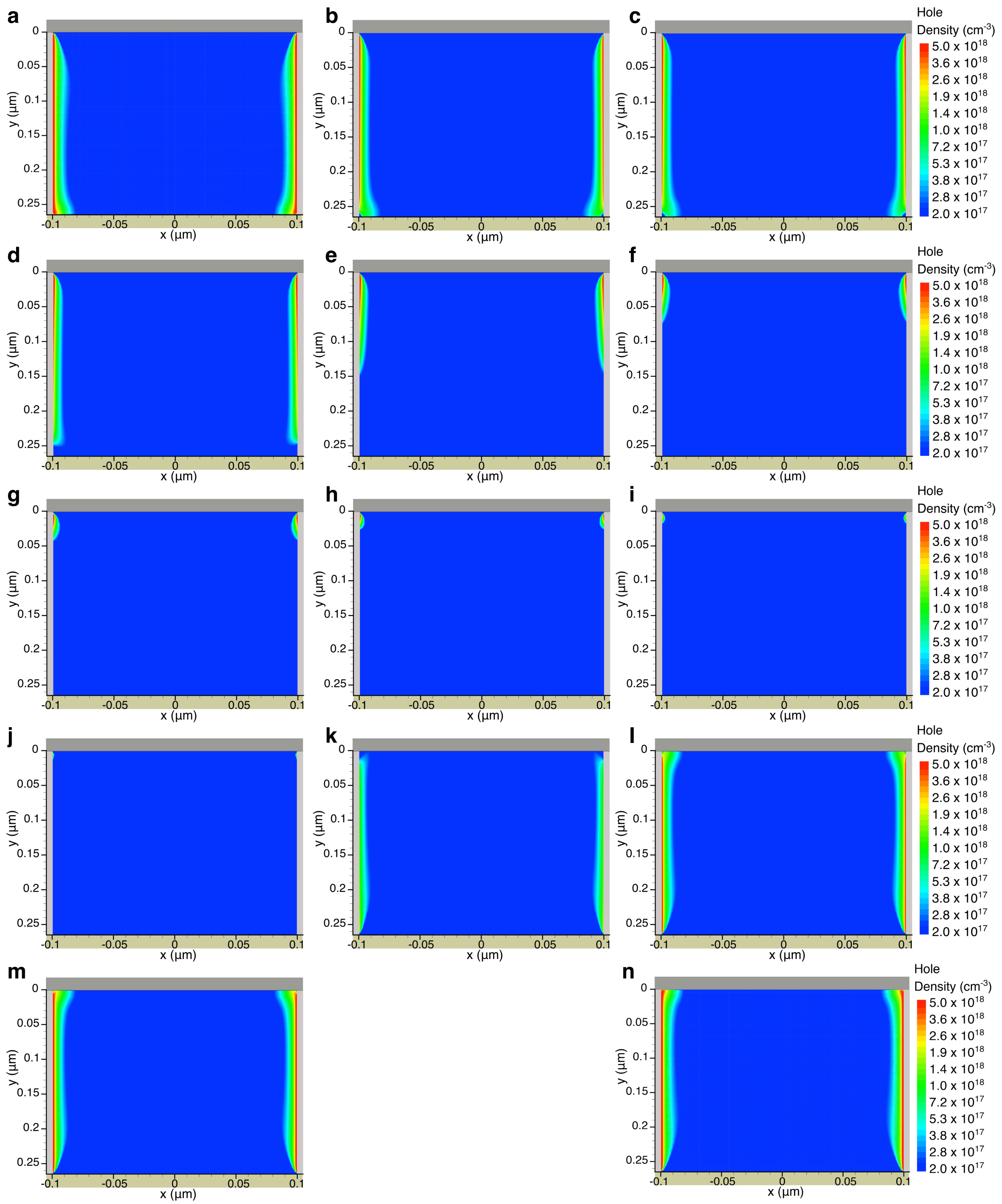

Supplementary Figure 9 | 2D distribution of the hole concentration. Calculated hole density into the PFO semiconductor as a function of the applied voltage. The current-voltage conditions are shown with symbols in Figure 8. (**a**) *V* = -20 V, (**b**) *V* = -5 V, (**c**) *V* = -3.75 V, (**d**) *V* = -2.5 V, (**e**) *V* = -1.25 V, (**f**) *V* = 0 V, (**g**) *V* = 1.25 V, (**h**) *V* = 2.5 V, (**i**) *V* = 3.75 V, (**j**) *V* = 5 V, (**k**) *V* = 12.5 V, (**l**) *V* = 13.75 V, (**m**) *V* = 15 V, (**n**) *V* = 20 V.

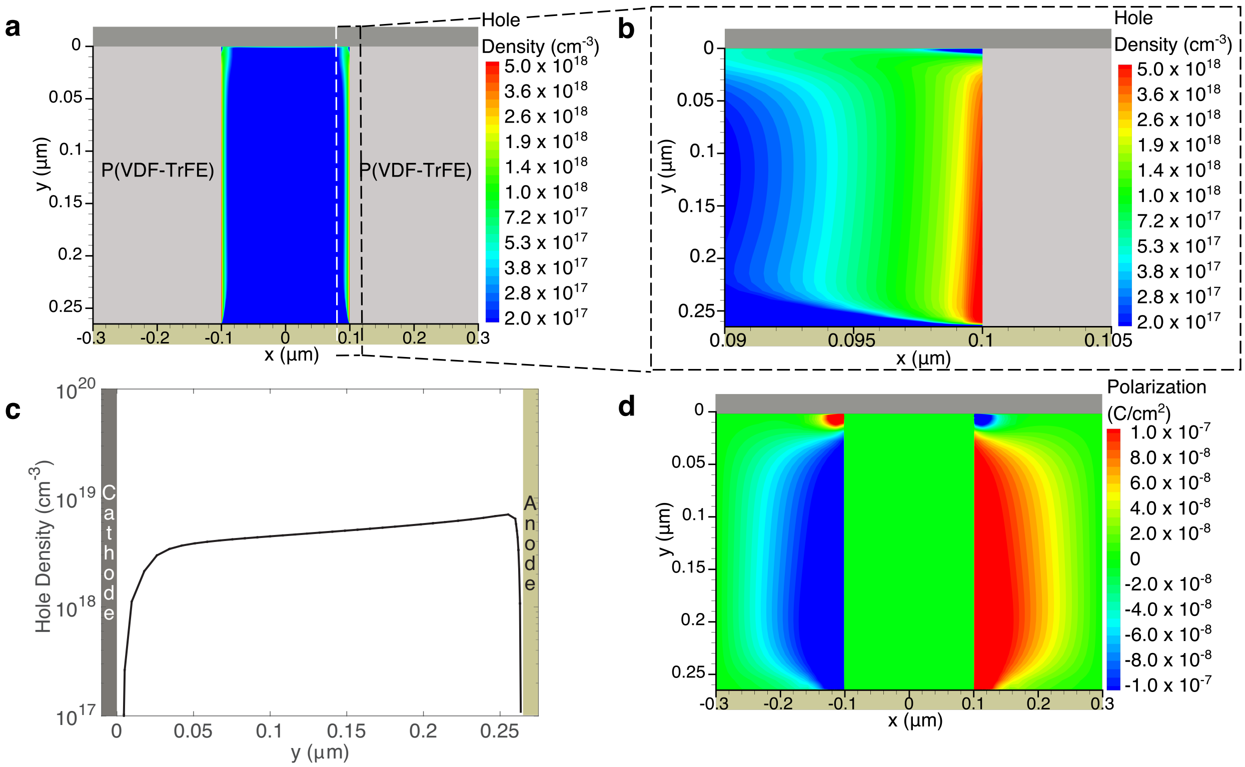

Supplementary Figure 10 | Operation of organic ferroelectric memory diodes. (**a**) Hole density distribution in the PFO pillar at *V* = 5 V. (**b**) Zoom of the hole density distribution at the right PFO/P(VDF-TrFE) interface. (**c**) Hole density along the PFO/P(VDF-TrFE) interface (cut line at *x* = 0.099 μm), *V* = 5 V. (**d**) x-component of the polarization vector at *V* = 5 V.

Supplementary Note 7| Further explanations on the x-polarization in P(VDF-TrFE)

The lateral *x*-polarization component is crucial for understanding the device physics. In a parallel-plates capacitor with P(VDF-TrFE) stacked between two electrodes, perfectly parallel field lines along *y*-direction occur without any lateral contribution. In a ferroelectric memory diode the situation is different. Due to the strong charge accumulation and the efficient tunneling, a potential equal to the electrode potential is set into the semiconductor (viz. the quasi-Fermi potential close to the contact is equal to the contact potential). As a consequence, the field lines are no longer along the *y*-direction, but are slightly bent and exhibit a non-zero *x*-component.

Supplementary Figure 11 shows the *x*-polarization in the P(VDF-TrFE) domain surrounded by two PFO pillars. The *x*-polarization is perfectly symmetric with respect to the central vertical line of the P(VDF-TrFE) domain. Although the *x*-polarization component is more than an order of magnitude lower than the vertical *y*-polarization, charge carriers are accumulated at the ferroelectric/semiconductor interfaces and, as shown in Figure 3e of the main manuscript, the magnitude of the on-current strongly depends on the *x*-polarization.

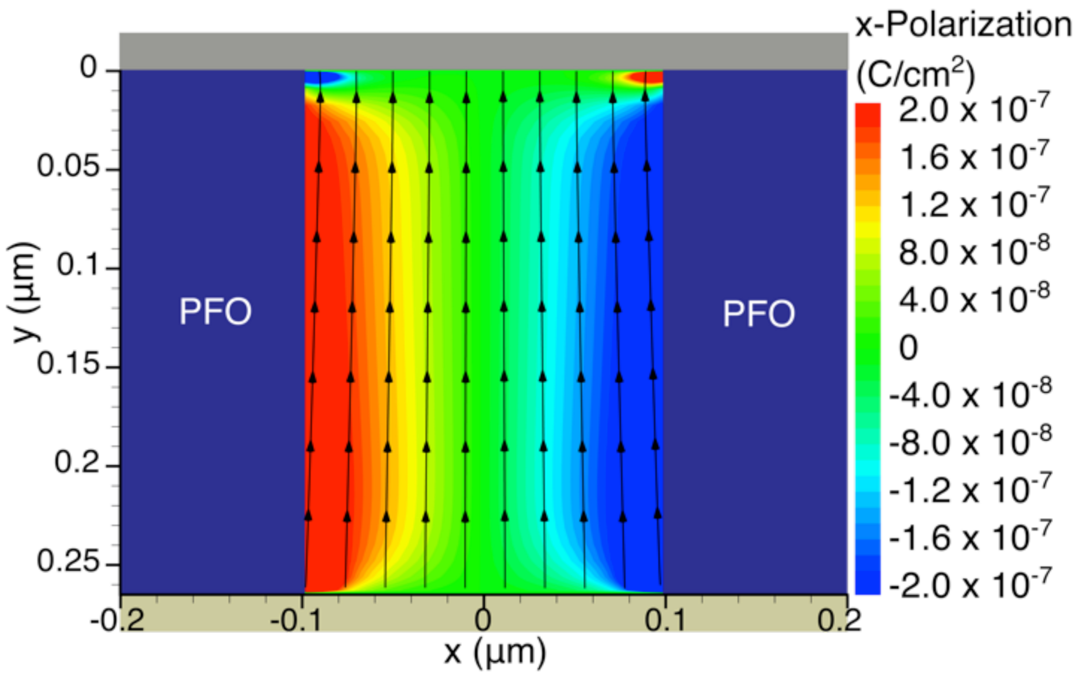

Supplementary Figure 11 | Electric field and polarization in P(VDF-TrFE). The *x*-polarization in the P(VDF-TrFE) domain (centre) is shown as color code. The electric field lines are represented by the black arrows.

Supplementary Note 8| Ferroelectric memory diodes with various thickness

The numerical simulations predict that the *I-V* characteristics of ferroelectric memory diodes are similar to those of a field-effect transistor operating in saturation at the pinch-off. Hence, the current at a given voltage is expected to be inversely proportional to the film thickness (L). In order to verify this prediction, we fabricated memory diodes, with Au bottom and top contacts, and a thickness between 300 nm and 500 nm. Supplementary Figure 12a shows a typical *I-V* curve for each device thickness. By varying L we found that the shape of the *I-V* characteristics is similar and only the magnitude of the current changes. We extracted the average current at 10 V from at least six devices for each thickness. The values are listed in Supplementary Table 3.

In order to obtain the effective interface length we analyzed the morphology with AFM. For thick films, not all the PFO domains are bicontinuous. Therefore, we selectively dissolved PFO by immersing the blend film in toluene under vigorous stirring at high temperatures. Toluene is a good solvent of PFO but not for P(VDF-TrFE). AFM height micrographs before and after selective dissolution are presented in Supplementary Figure 13. The value for the extracted effective interface length is presented in Supplementary Table 3.

The effective on-current (viz. the on-current scaled by the interface length) is presented in Figure 2f in the main manuscript, here reproduced as Supplementary Figure 12b. The memory diode on-current at V = 10 V scales linearly with the reciprocal layer thickness, and this is in perfect agreement with numerical calculations.

Supplementary Table 3 | Morphological and electrical parameters for memory diodes with various thickness. The presented values are average values based on the electrical characterization of at least six diodes for each thickness. The interface length is the average value of at least 6 different AFM micrographs measured at different positions. In order to determine the interface length, PFO was selectively dissolved.

Thickness	487± 4 nm	399± 6 nm	314 ± 8 nm
Current at 10V	1.21 ± 0.04 μA	4.1 ± 0.7 μA	9.6 ± 0.9 μA
Interface length on 5x5 μm^2	2.34 ± 0.7 μm	6.5 ± 0.6 μm	11.0 ± 2 μm
Current / interface length	81 μA/m	96 μA/m	136 μA/m

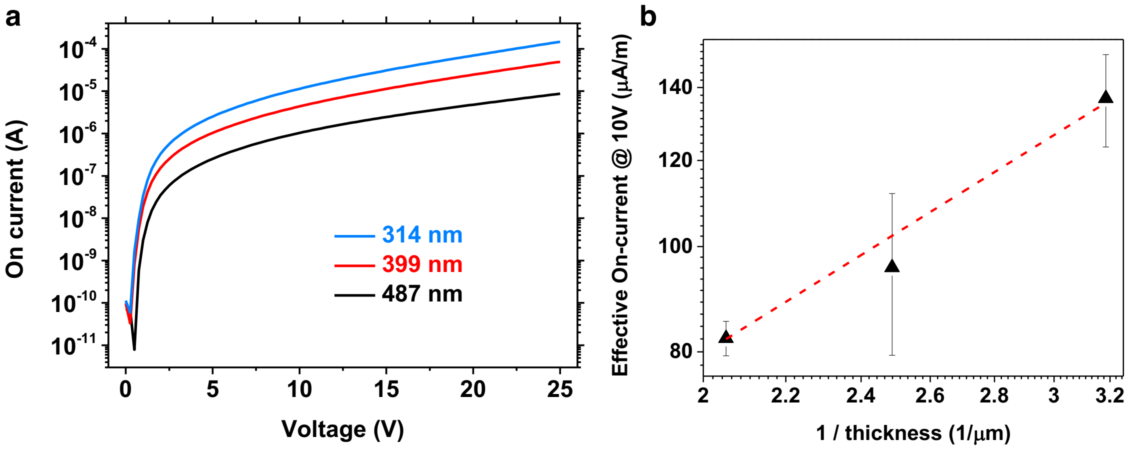

Supplementary Figure 12 | Thickness dependent *I-V* characteristics of ferroelectric memory diodes. (**a**) On-current as function of applied bias for memory diodes with various thickness of the P(VDF-TrFE)/PFO blend film. (**b**) On-current normalized by the effective interface length as a function of the reciprocal layer thickness. The dashed line is the linear least square approximation. Error bars are the standard deviation.

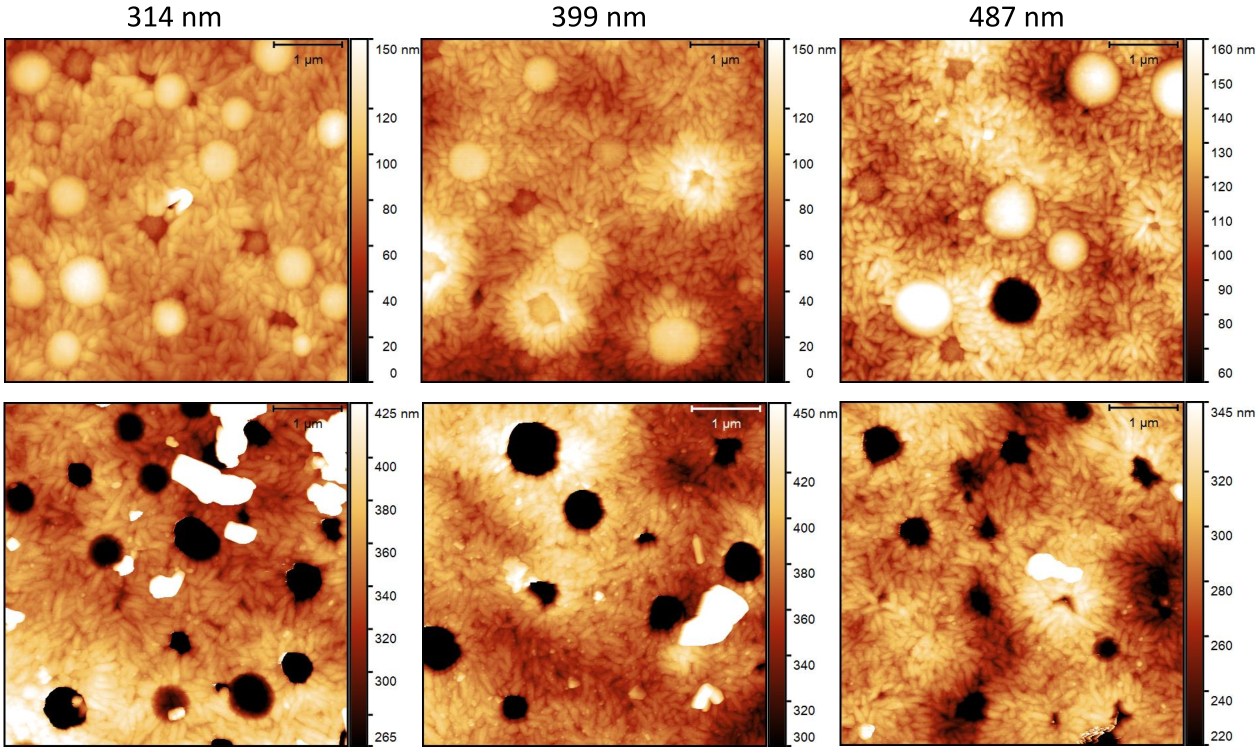

Supplementary Figure 13 | Blend morphology before and after selective dissolution of PFO for different film thickness. AFM height micrographs before (first row), and after (second row) selective dissolution of PFO. Since toluene is a good solvent for PFO but does not dissolve P(VDF-TrFE), PFO could be selectively dissolved from the blend films by immersing the substrate in toluene under vigorous stirring at high temperatures.

Supplementary Note 9| Impact of the tunnelling and doping on the off-current

Supplementary Figure 14 shows the *I-V* characteristics of ferroelectric memory diodes calculated with (full lines) and without (dashed lines) the tunnelling injection as a function of the unintentional background doping $N_D = \{10^{15}, 10^{16}, 5\ 10^{16}, 10^{17}\}$ cm^{-3}. In both cases (i.e. with and without tunnelling) the on-current is independent of the doping concentration whereas the off-current depends on the doping concentration and, as expected, it increases by increasing the doping. More in detail, Supplementary Figure 15 shows the ratio between the off-current with and without the tunnelling current (I_{wT}/I_{woT}) at V = 5 V as a function of N_D. $I_{wT}/I_{woT} = 6$ at $N_D = 10^{15}$ cm^{-3} and $I_{wT}/I_{woT} > 40$ at $N_D = 10^{17}$ cm^{-3}, thus confirming that the tunnelling current is relevant also when the ferroelectric memory diodes operate in the off-state. Since the injection barrier is high (in our case Φ_{B0} = 1.3 eV), it is reasonable that the tunnelling current is the main contribution also in the off-state. To gain more insight on the effect of the background doping on the off-state tunnelling current, Supplementary Figure 16 shows the tunnelling rate distribution within the PFO semiconductor in the cases $N_D = 10^{15}$ cm^{-3} (panel a) and $N_D = 10^{17}$ cm^{-3} (panel b), at V = 5 V. The tunnelling takes place through about 4.5 nm and 3 nm and the maximum rate is about 3 10^{16} cm^{-2} s^{-1} and 1 10^{19} cm^{-2} s^{-1}, respectively. Interestingly, Supplementary Figure 17a shows that the tunnelling rate in the off-state is more than five order of magnitude lower than the tunnelling rate in the on-state, but it is still sufficient to sustain the off-current. In addition, Supplementary Figure 17b shows that the tunnelling distance increases by decreasing the doping concentration. The maximum distance is 4.5 nm when $N_D = 10^{15}$ cm^{-3} and the minimum distance is 3 nm when $N_D = 10^{17}$ cm^{-3}. It is worth noting that the aforementioned tunnelling distances are comparable with those obtained in coplanar organic transistors.[45]

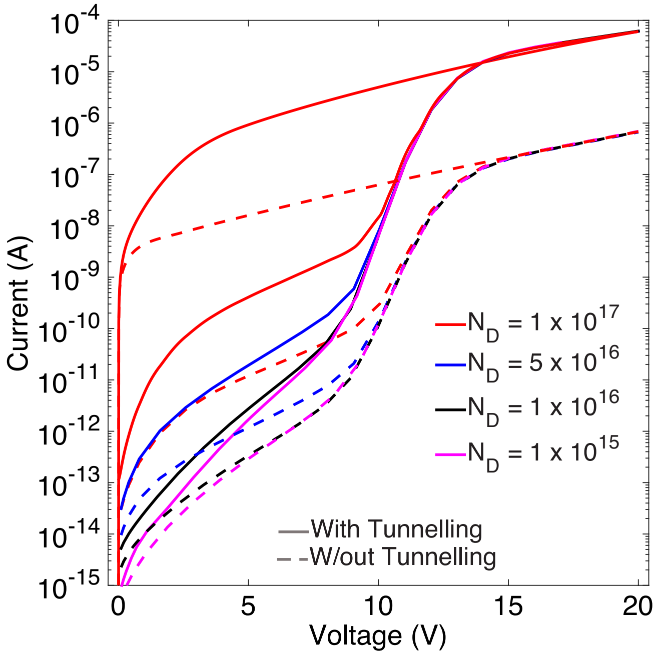

Supplementary Figure 14 | Impact of the tunnelling injection and unintentional doping on the memory diode characteristics. *I-V* characteristics calculated with (full line) and without (dashed line) tunnelling injection and varying the unintentional background doping N_D.

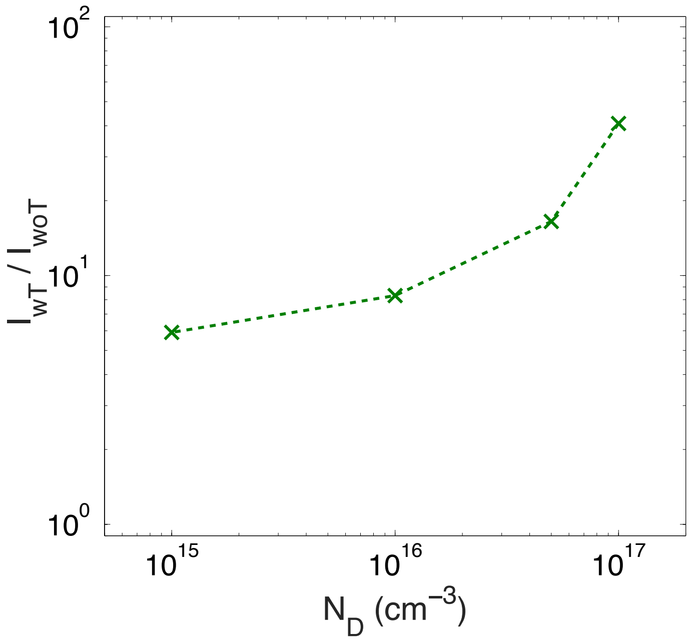

Supplementary Figure 15 | Impact of the unintentional doping and tunnelling on the off-current. Ratio between the off-current with (I_{wT}) and without (I_{woT}) tunnelling at V = 5 V as a function of the unintentional doping concentration N_D.

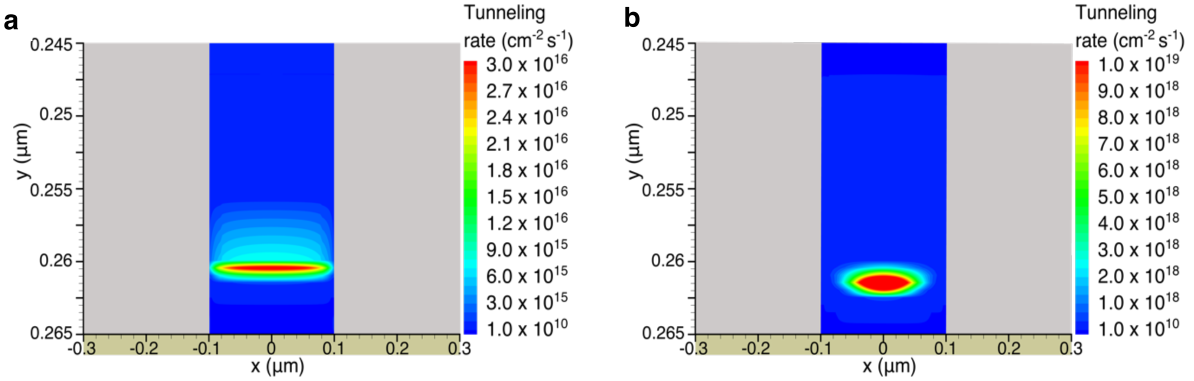

Supplementary Figure 16 | 2D distribution of the tunnelling rate in a memory diode in the off-state. Tunnelling rate distribution within the PFO semiconductor bias at V = 5 V. (**a**) N_D = 10^{15} cm^{-3}. (**b**) N_D = 10^{17} cm^{-3}.

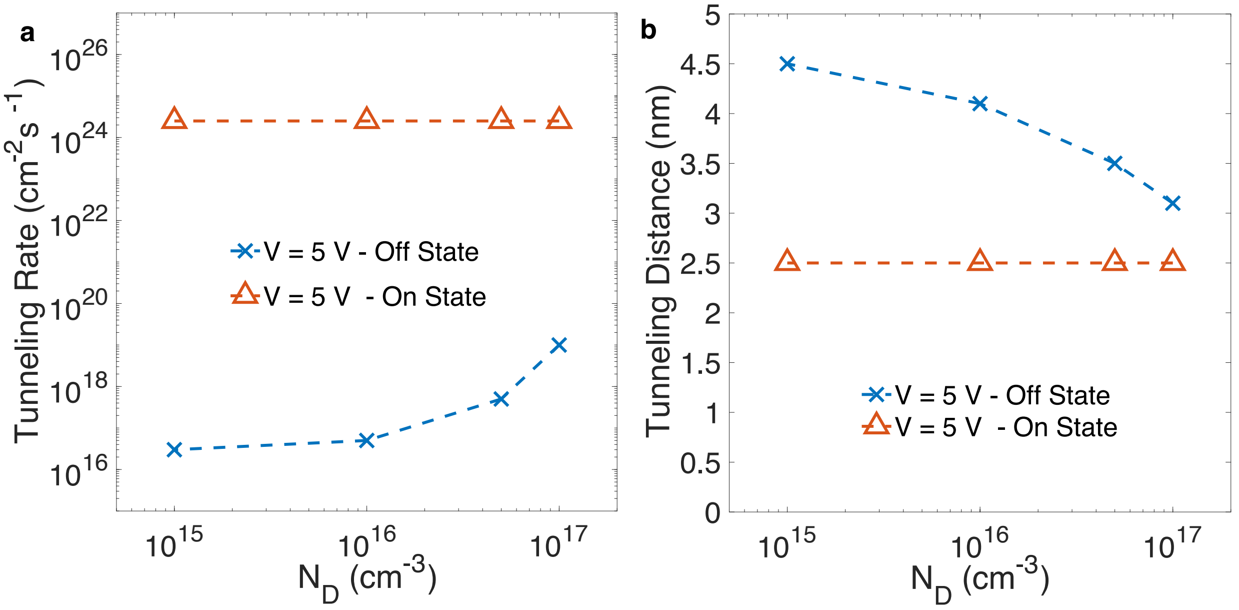

Supplementary Figure 17 | Impact of the unintentional doping on the tunnelling rate and tunnelling distance. (**a**) Tunnelling rate and (**b**) tunnelling distance as a function of the doping concentration.

Supplementary Note 10| Downscaling the feature size of the PFO pillar

Figure 3d in the main manuscript shows that the calculated hole-only diode current (dashed line) is comparable with the current measured in ferroelectric memory diodes operated in the on-state (i.e. the ferroelectric is polarized). In Figure 3d (x symbols) we plot the measurement of the hole-only diode normalized by the proper normalization factor. The normalization factor ($N_F = G_F \times M_F \times T_F$) accounts for the different surface area and thickness in the hole-only diode and the ferroelectric diode. The surface area accounts for both the geometrical and morphological area. The geometrical factor is calculated as $G_F = A_{memory\text{-}diode} / A_{hole\text{-}only} = 0.02$, where $A_{memory\text{-}diode} = 0.16$ mm^2, $A_{hole\text{-}only} = 8$ mm^2. The morphological factor (M_F) is calculated as follows. According with the AFM micrographs of ferroelectric memory diodes presented in Supplementary Figure 5, the area of the PFO domains is 1.7 μm^2 in a scanned area of 25 μm^2, viz. only the 7 % of the total area, and therefore $M_F = 0.07$. This is expected since the memory diodes are fabricated using a solution that contains 10% of PFO with respect to the entire polymer weight. Moreover, the thickness factor is calculated as $T_F = (L_{hole\text{-}only} / L_{memory\text{-}diode})^3 = 0.5$, where $L_{memory\text{-}diode}$ = 265 nm, $L_{hole\text{-}only}$ = 210 nm, and the power 3 accounts for the space charge limited transport in hole-only diodes. Therefore, the current measured in hole-only diodes can be compared with the current of ferroelectric diodes by using a normalization factor equal to $N_F = G_F \times M_F \times T_F = 7\ 10^{-4}$.

In addition, we note that if the PFO feature size (W_{PFO}) is scaled down, the ferroelectric memory diode current is preserved, while the corresponding PFO hole only diode current linearly drops. As an example, Supplementary Figure 18 shows that when W_{PFO} = 5 nm the current of the ferroelectric memory diode exceeds that of the corresponding PFO hole-only diode by about one order of magnitude.

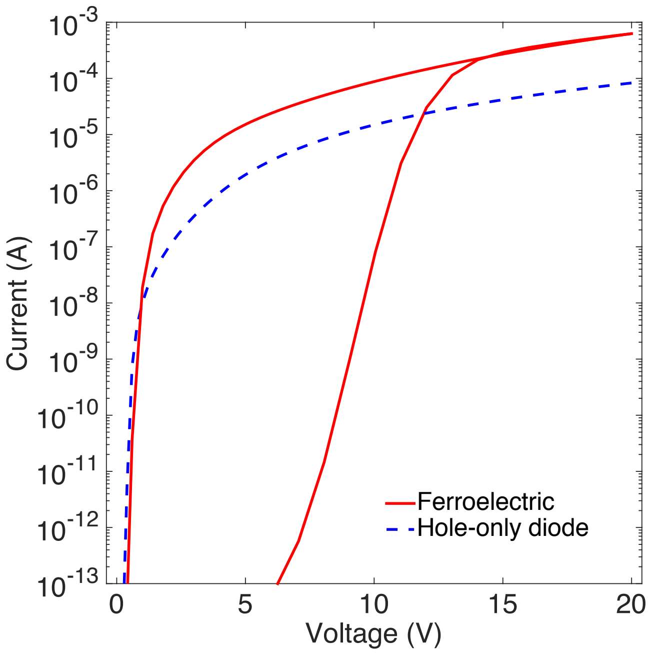

Supplementary Figure 18 | Impact of the semiconductor feature size. Calculated *I-V* characteristics of a hole-only diode (dashed line) and ferroelectric memory diode (full line) when the feature size W_{PFO} = 5 nm.

Supplementary Note 11| Further experimental investigations on the interface transport

We performed conductive AFM measurements on one pillar of PFO surrounded by P(VDF-TrFE). We prepared a 50 nm thick P(VDF-TrFE):PFO blend film deposited on glass slides fully coated with Cr+Au. Cr served as adhesion layer while Au is the bottom electrode of the memory diodes. The P(VDF-TrFE):PFO blend film was deposited by wire-bar coating. We deliberately carried out the processing in the same way as for the fabricated memory diodes in the manuscript (Method Section). Here, only the film thickness is lower (50 nm instead of 265 nm) in order to achieve the poling with the conductive AFM, where the maximum voltage is limited to 10 V (limited bias range in the tool). To decrease the film thickness resulting from wire-bar coating, we decreased the concentration of the polymer mixture dissolved in cyclohexanone from 45 mg/ml (in the main manuscript) to 20 mg/ml.

As measurement tool, we used a Dimension Icon FS from Bruker (electrical mode: PeakForce Tuna; cantilever type: Brucker SCM-PIT; resonant freq: 75 kHz; spring const.: 2.8 N/m; tip model OTESPA-R3: tip coating: conductive Pt/ Ir). The Pt/Ir coated tip serves as top contact for the memory diode.

Supplementary Figure 19a shows an example of an AFM topography measurement. A semiconducting PFO domain of about 250 nm diameter is embedded in a matrix of the ferroelectric polymer P(VDF-TrFE). The topography and the current were measured simultaneously and Supplementary Fig. 19b shows the current through the film when the applied voltage is +10 V. The bias is applied to the bottom Au contact (injecting electrode) with respect to the top (extracting) electrode, i.e. the cantilever. Since the film thickness is only 50 nm, the coercive voltage is about 2 V and hence the applied voltage (10 V) is suitable to pole the P(VDF-TrFE). According with the numerical simulations, we expect hole injection from the Au bottom contact and current flow through the PFO at the ferroelectric/semiconductor interface. This is confirmed by Supplementary Fig. 19b where the high current flow is indicated by bright regions directly at the interface between PFO and P(VDF-TrFE).

We note that a quantitative analysis with conductive AFM requires careful analysis. The curvature of the tip is typically 5-10 nm, thus fundamentally limiting the lateral resolution to at best 5-10 nm. Moreover, ferroelectric memory diodes are obtained by spontaneous phase separation between the semiconducting PFO and the ferroelectric P(VDF:TrFE) polymers. This hampers the formation of extremely sharp PFO/P(VDF:TrFE) interfaces (viz. Supplementary Fig. 6), thus yielding to a non-uniform distribution of the current around the PFO domain. Finally, the conductive AFM operation is based on application of the mechanical force on the tip in contact with the film. Since P(VDF-TrFE) is a piezoelectric material, application of such forces leads to piezoelectric voltage due to direct piezoelectric effect. Hence a detailed analysis requires decoupling of the piezoelectric response. For instance, we found that the force applied to the surface with the cantilever (deflection set-point) during the measurement strongly impacts the measured current. Providing a detailed profile of the current distribution within the PFO pillar or disentangling the mechanical and electrical contributions is beyond the scope of this work.

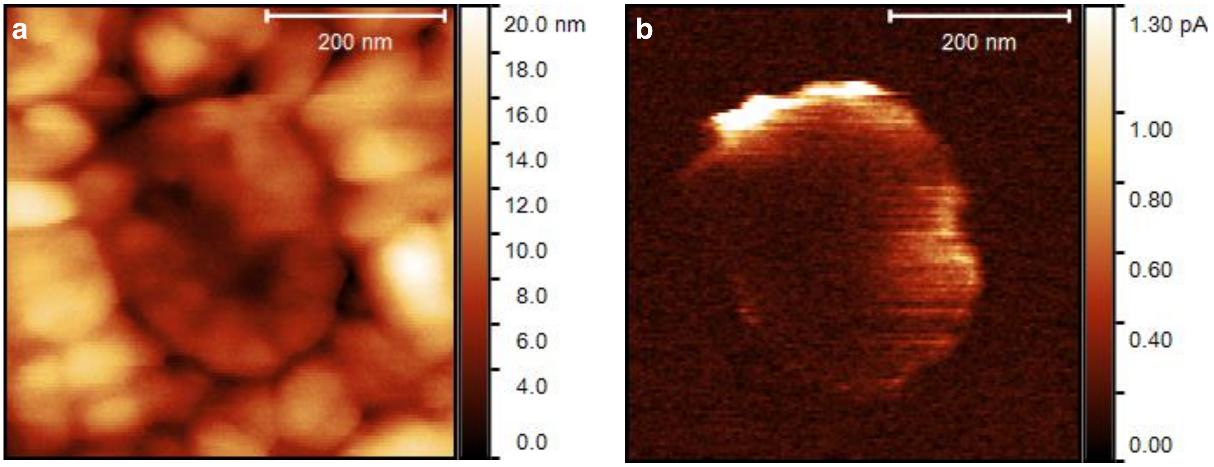

Supplementary Fig. 19 | Conductive AFM analysis of a PFO pillar. (**a**) AFM topography image of a phase separated P(VDF-TrFE):PFO film on Au. (**b**) Current measured when applying +10 V at the bottom contact (Au) with respect to the AFM tip (cantilever).

Supplementary Note 12 | Downscaling the feature size of the P(VDF-TrFE) domain

In ferroelectric memory diodes, the charge transport takes place in 2.5 nm wide channels, and hence the PFO domain diameter can be scaled down to 5 nm with almost no impact on the on-current. In order to further enhance the storage density, we analyze the impact of downscaling the lateral dimension of the P(VDF-TrFE) domains on the charge accumulation and, in turn, on the on-current.

Supplementary Figure 20a (left panel) shows the charge density in the PFO pillar (W_{PFO} = 5 nm) when the PFO domain is surrounded by P(VDF-TrFE) domains with a width of $W_{P(VDF\text{-}TrFE)}$ = 200 nm. Two strongly accumulated hole channels fill the whole PFO domain because of the lateral polarization in P(VDF-TrFE) illustrated in Supplementary Figure 20a (right panel).

When the P(VDF-TrFE) domain size is decreased to $W_{P(VDF\text{-}TrFE)}$ = 50 nm (Supplementary Figure 20b), the *x*-polarization decreases as well, leading to a lower charge accumulation in the PFO pillar. In the case of smaller P(VDF-TrFE) domains, the *x*-polarization component occurs only in a portion of the ferroelectric domain ($W_{P(VDF\text{-}TrFE)}$ = 10 nm, Supplementary Figure 20c). If $W_{P(VDF\text{-}TrFE)}$ = 5 nm and W_{PFO} = 5 nm (Supplementary Figure 20d), the *x*-polarization is extremely reduced. Hence, the charge carrier density is more than one order of magnitude lower compared to the case $W_{P(VDF\text{-}TrFE)}$ = 200 nm.

It should be noted that the on-current of the memory diode in the case of $W_{P(VDF\text{-}TrFE)}$ = 5 nm and W_{PFO} = 5 nm is equivalent to the case of $W_{P(VDF\text{-}TrFE)}$ = 200 nm and W_{PFO} = 200 nm. Although the memory diodes have the same area (0.16 mm^2), in the former case ($W_{P(VDF\text{-}TrFE)}$ = 5 nm and W_{PFO} = 5 nm) the total interface length is larger because of the larger number of PFO pillars. This compensates the lower accumulation due to the reduced *x*-polarization. As a consequence, the high current density in the on-state can be maintained upon downscaling.

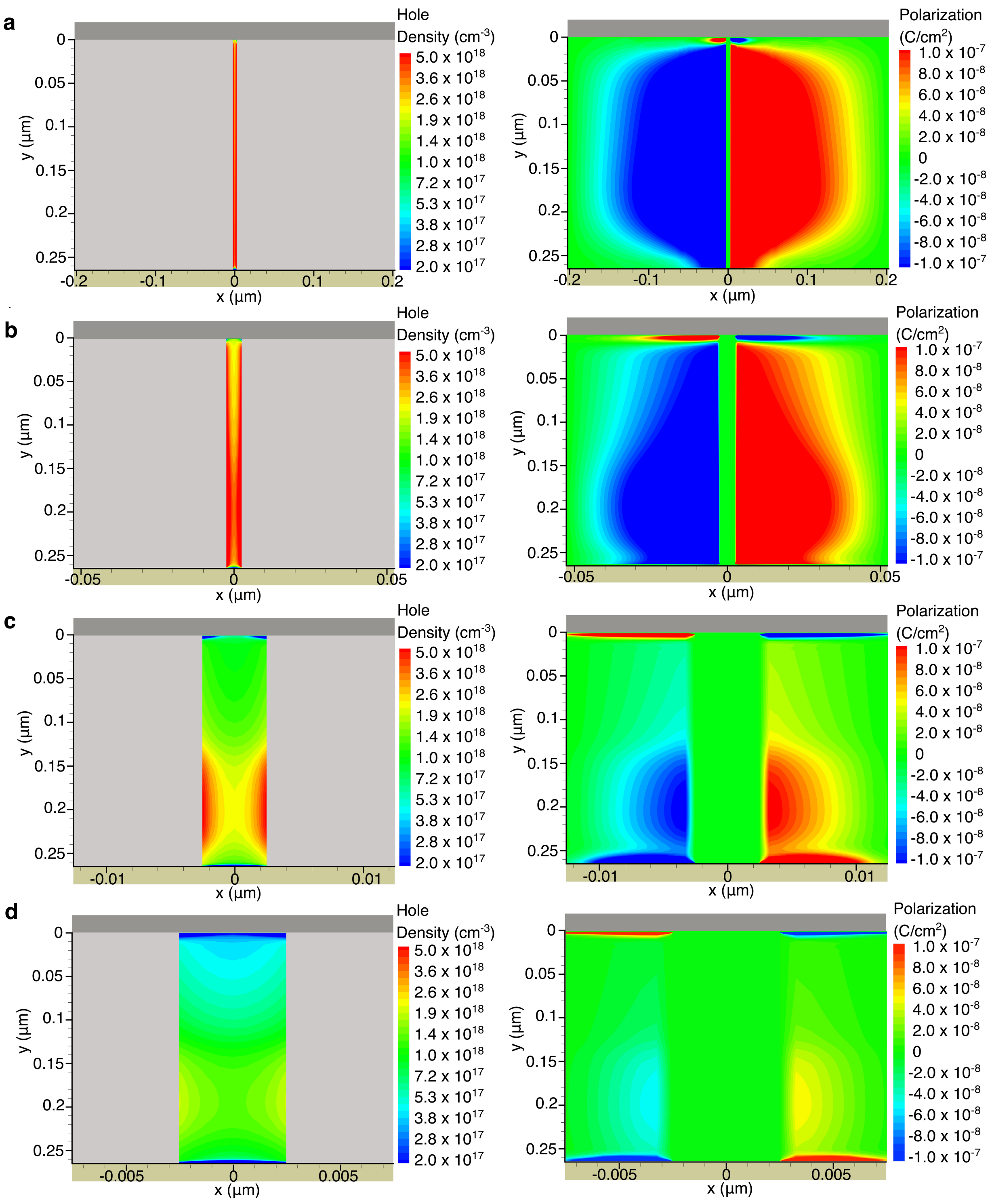

Supplementary Figure 20 | Scaling of the P(VDF-TrFE) domain. Hole density distribution in the PFO pillar (left panels) and *x*-component of the polarization vector (right panels) at $V = 5$. The lateral dimension of the PFO pillar is $W_{PFO} = 5$ nm. The lateral dimension of the P(VDF-TrFE) domain are: (**a**) $W_{P(VDF\text{-}TrFE)} = 200$ nm, (**b**) $W_{P(VDF\text{-}TrFE)} = 50$ nm, (**c**) $W_{P(VDF\text{-}TrFE)} = 10$ nm, and (**d**) $W_{P(VDF\text{-}TrFE)} = 5$ nm.

Supplementary Note 13 | Analysis of the cross-talk

To evaluate the effect of the cross-talk on aggressively scaled ferroelectric memory diodes, we simulated three adjacent memory diodes separated by 5 nm as shown in Supplementary Figure 21.

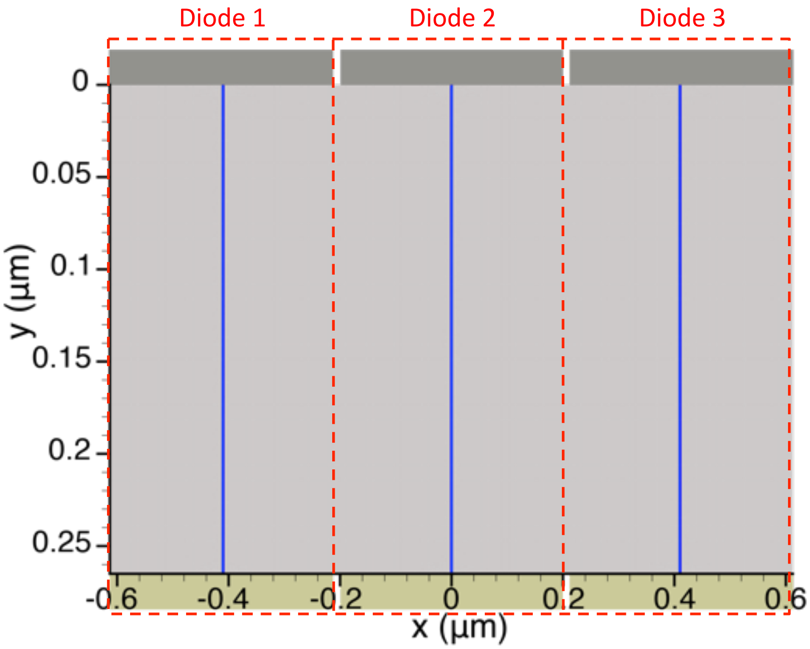

Supplementary Figure 21 | Cross-section of three adjacent memory diodes. The dashed rectangles highlight the three memory diodes. The blue rectangles are the semiconducting PFO pillars while the light gray rectangles are the PVDF-TrFE ferroelectric polymer. For each memory diode the lateral dimension of the PFO is set to the minimum theoretical limit W_{PFO} = 5 nm, and the lateral dimension of the P(VDF-TrFE) is $W_{P(VDF-TrFE)}$ = 200 nm.

All the top contacts (dark gray rectangles) are biased at 0 V, the lateral bottom contacts (gold rectangles) at left and right (diodes 1 and 3) are biased at 0.1 V while the voltage of the central bottom contact (gold rectangle, diode 2) is swept from 0 V to positive voltages. Therefore, the memory diodes 1 and 3 are in the off-state and they are not programmed whereas the memory diode 2 is programmed. The lateral dimension of PFO is set to the minimum theoretical limit W_{PFO} = 5 nm, the lateral dimension of the P(VDF-TrFE) is varied as follows $W_{P(VDF-TrFE)}$ = {200, 10, 5} nm and the area factor is calculated accordingly. Supplementary Figure 22 shows the calculated I-V characteristics of the memory diode 2 as a function of $W_{P(VDF-TrFE)}$ scaling. The current of the memory diodes 1 and 3 is always below 10^{-15} A. When $W_{P(VDF-TrFE)}$ = 200 nm the maximum current of the memory diode 2 is larger than 10^{-2} A. Strikingly, comparing the current of the diode 2 shown in Supplementary Figure 22 (red full line) with that of a single diode shown in Figure 4b (blue full line, main manuscript), the former is about one order of magnitude larger. On the other hand, when $W_{P(VDF-TrFE)}$ is 10 nm and 5 nm the current of the diode 2 decreases (Supplementary Figure 22, blue and black lines) and does not show the typical hysteretic loop, i.e. a memory behavior. We note that when we considered only one diode, we found a very nice memory behavior even when

$W_{P(VDF\text{-}TrFE)}$ = 5 nm. Both the improved on-current and the lack of the memory behavior in the characteristics presented in Supplementary Figure 22 are related to the cross-talk between adjacent memory diodes.

To gain more insight on this important point, Supplementary Figure 23 shows the *y*-component of the polarization vector, the electric field stream lines and the hole density in the cases $W_{P(VDF\text{-}TrFE)}$ = 200 nm (panels a,b) and $W_{P(VDF\text{-}TrFE)}$ = 5 nm (panels c,d).

When $W_{P(VDF\text{-}TrFE)}$ = 200 nm, most of the electric field lines start from the central bottom contact (diode 2, biased at 20 V) and end at the top contacts (biased at 0 V), while only few of them start from the central bottom contact and end at the lateral bottom contacts of the diodes 1 and 3. The ferroelectric polymer around the diode 2 is fully polarized (Supplementary Figure 23a). As a consequence, the channels in the diode 2 are accumulated (Supplementary Figure 23b), and no channels are formed in the diodes 1 and 3. Moreover, the lateral diodes (1 and 3) increase the electric field near the injecting contact and this, in turn, results in a larger on-current of the diode 2 compared to that of a single diode shown in Figure 4b (blue full line).

In contrast, in the case $W_{P(VDF\text{-}TrFE)}$ = 5 nm (Supplementary Figure 23c) most of the electric field lines start from the central bottom contact and end at lateral bottom contacts. As a consequence, the diode 2 shows weak channel accumulations (Supplementary Fig 23d). This can be explained as follows. Since the distance between the diodes is 5 nm while the diodes thickness is L = 265, the horizontal electric field (F_x) in the neighborhood of the injecting bottom contact of the diode 2 is much larger the vertical electric field (F_y). Therefore, the ferroelectric polymer gets fully polarized only near the bottom contact of diode 2. The memory diode 2 shows an extremely limited hysteresis, and the memory effect is vanished. In this case, the cross-talk prevents the diode 2 operation while the diodes 1 and 3 are not affected.

In order to limit the cross-talk between aggressively scaled ferroelectric memory diodes, it is necessary to scale down the diode thickness (L), too. It is worth noting that L = 50 nm can be easily obtained in phase separated memory diodes, as reported in the Supplementary Figure 7. Supplementary Figure 24 shows the current of diode 2 in the case $W_{P(VDF\text{-}TrFE)}$ = 5 nm, when L = 265 nm (black line) and L = 50 nm (magenta line). Here the hysteresis (the memory behavior) is restored and at a reading voltage of 1V the on/off current ratio is of about 10^6. The cross-talk can be disregarded. This is further confirmed in Supplementary Figure 25. Despite that several electric field lines start from the central bottom contact and end at the lateral bottom contacts, the ferroelectric polymer around the central diode is significantly polarized (Supplementary Figure 25a), and the injected holes are transported through the channels accumulated in the memory diode 2 (Supplementary Figure 25b).

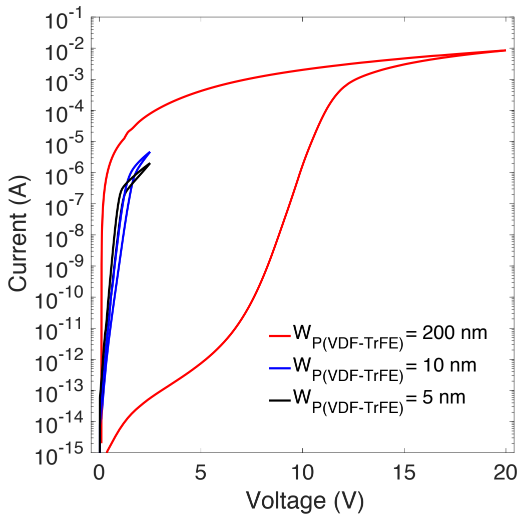

Supplementary Figure 22 | Impact of the downscaling on the cross-talk. *I-V* characteristics of the memory diode 2 at several $W_{P(VDF-TrFE)}$. The thickness is L = 265 nm. The calculated current of the lateral diodes (left and right) is lower than 10^{-15} A.

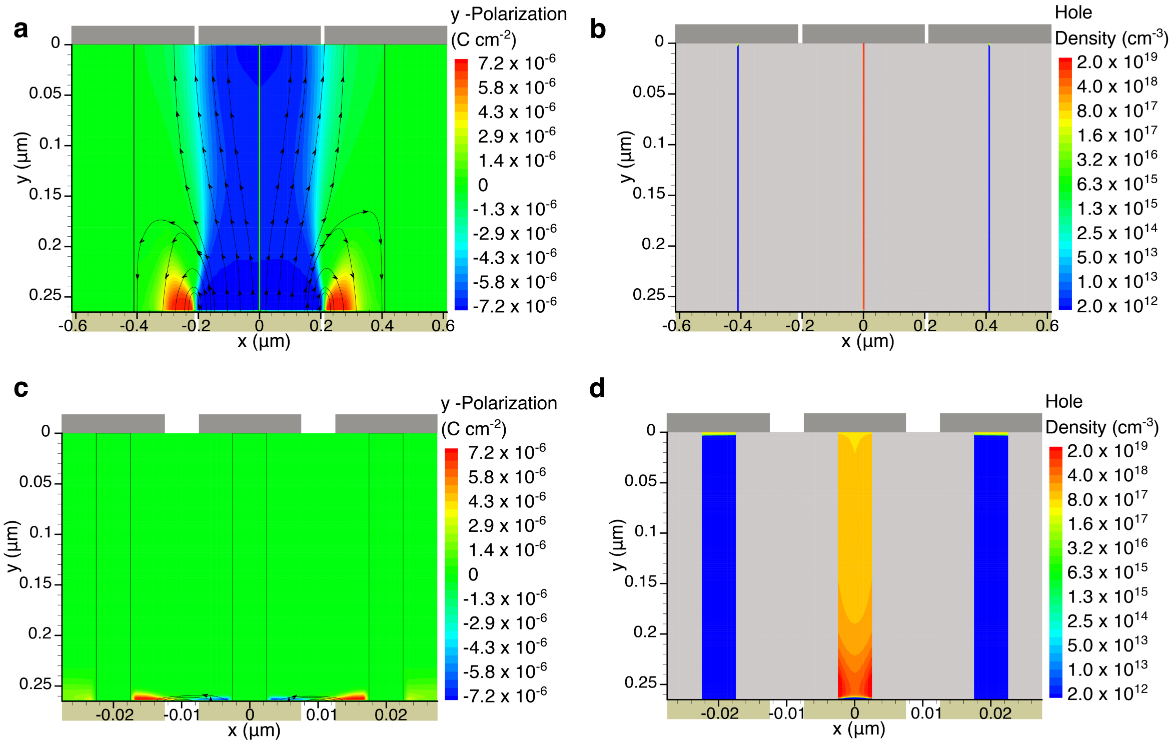

Supplementary Figure 23 | Polarization and hole density in adjacent memory diodes by scaling the ferroelectric lateral dimension. 2D numerical simulations of y-polarization component and hole density distribution in three adjacent ferroelectric memory diodes with thickness L = 265 nm. The stream lines indicate the electric field distribution within the memory diodes. The top contacts are biased at 0 V, the left and right bottom contacts (diodes 1 and 3) are biased at 0.1 V, the central bottom contact (diode 2) is biased at 20 V. (**a**) and (**b**) $W_{P(VDF-TrFE)}$ = 200 nm. (**c**) and (**d**) $W_{P(VDF-TrFE)}$ = 5 nm.

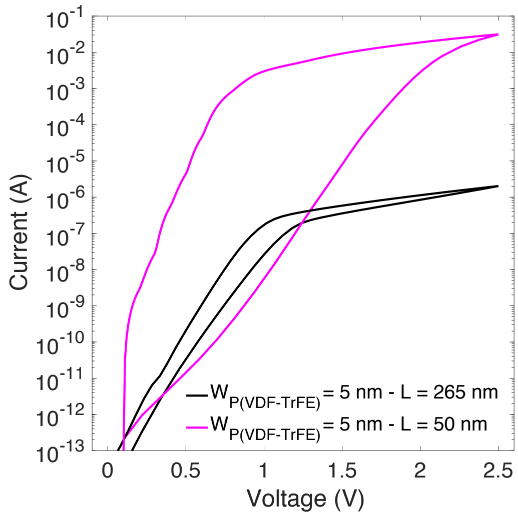

Supplementary Figure 24 | Impact of the downscaling on the cross-talk. *I*-*V* characteristics of the memory diode 2 at several thicknesses (*L*). In all cases $W_{\mathrm{P(VDF\text{-}TrFE)}}$ = 5 nm.

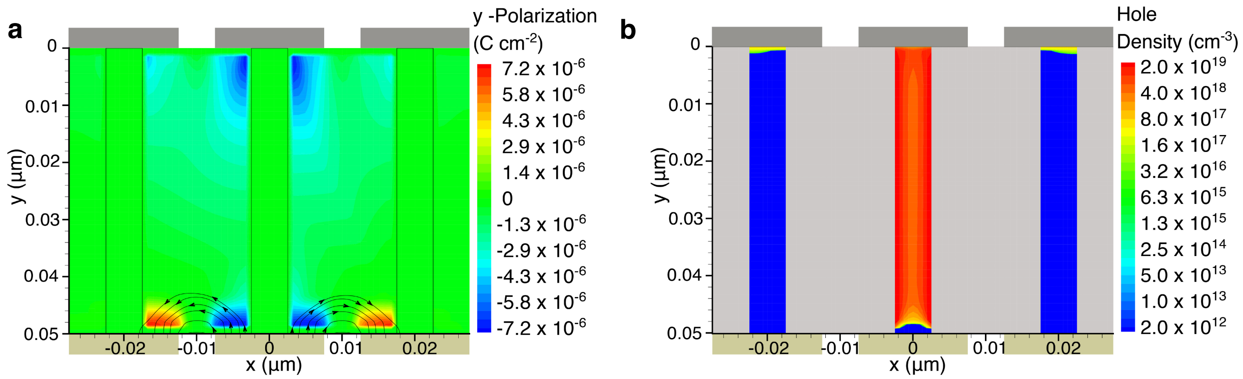

Supplementary Figure 25 | Polarization and hole density in adjacent aggressively scaled memory diodes. 2D numerical simulations of (**a**) y-polarization and (**b**) hole density distribution in three adjacent ferroelectric memory diodes with thickness *L* = 50 nm and $W_{\mathrm{P(VDF\text{-}TrFE)}}$ = 5 nm. The stream lines indicate the electric field distribution within the memory diodes. The top contacts are biased at 0 V, the left and right bottom contacts (diodes 1 and 3) are biased at 0.1 V, the central bottom contact (diode 2) is biased at 2.5 V.

Supplementary References

1. Miller S. L. & McWhorter P. J. Physics of the ferroelectric nonvolatile memory field effect transistor. *J. Appl. Phys.* **72**, 5999-6010 (1992).

2 Miller S. L., Nasby R. D., Schwank J. R., Rodgers M. S. & Dressendorfer P. V. Device modeling of ferroelectric capacitors. *J. Appl. Phys.* **68**, 6463-6471 (1990).

3. Bo J, Zurcher P, Jones RE, Gillespie SJ, Lee JC. Computationally Efficient Ferroelectric Capacitor Model For Circuit Simulation. *Digest of Technical Papers* in *VLSI Technology,* (1997).

4. Dragosits K, Selberherr S. Two-dimensional simulation of ferroelectric memory cells. *IEEE T. Electron Dev.* **48**, 316-322 (2001).

5. Vissenberg MCJM, Matters M. Theory of the field-effect mobility in amorphous organic transistors. *Phys. Rev. B* **57**, 12964-12967 (1998).

6. Ambegaokar V., Halperin B. I. & Langer J. S. Hopping Conductivity in Disordered Systems. *Phys. Rev. B* **4**, 2612-2620 (1971).

7. Miller A, Abrahams E. Impurity Conduction at Low Concentrations. *Phys. Rev.* **120**, 745-755 (1960).

8. Coehoorn R., Pasveer W. F., Bobbert P. A. & Michels M. A. J. Charge-carrier concentration dependence of the hopping mobility in organic materials with Gaussian disorder. *Phys. Rev. B* **72**, 155206 (2005).

9. Torricelli F, Colalongo L, Milani L, Kovács-Vajna ZM, Cantatore E. Impact of energetic disorder and localization on the conductivity and mobility of organic semiconductors. In: *2011 International Conference on Simulation of Semiconductor Processes and Devices* (2011).

10. Pasveer W. F.*, et al.* Unified Description of Charge-Carrier Mobilities in Disordered Semiconducting Polymers. *Phys. Rev. Lett.* **94**, 206601 (2005).

11. van der Holst J. J. M.*, et al.* Modeling and analysis of the three-dimensional current density in sandwich-type single-carrier devices of disordered organic semiconductors. *Phys. Rev. B* **79**, 085203 (2009).

12. Ng T. N., Silveira W. R. & Marohn J. A. Dependence of Charge Injection on Temperature, Electric Field, and Energetic Disorder in an Organic Semiconductor. *Phys. Rev. Lett.* **98**, 066101 (2007).

13. Gartstein YN, Conwell EM. Field-dependent thermal injection into a disordered molecular insulator. *Chem. Phys. Lett.* **255**, 93-98 (1996).

14. Emtage P. R. & O'Dwyer J. J. Richardson-Schottky Effect in Insulators. *Phys. Rev. Lett.* **16**, 356-358 (1966).

15. Sze S, Ng KK. *Physics of Semiconductor Devices, Third Edition,* Wiley (2006).

16. Asadi K., de Boer T. G., Blom P. W. M. & de Leeuw D. M. Tunable Injection Barrier in Organic Resistive Switches Based on Phase-Separated Ferroelectric–Semiconductor Blends. *Adv. Funct. Mater.* **19**, 3173-3178 (2009).

17. MeiKei I, Solomon PM, Laux SE, Wong HSP, Chidambarrao D. Comparison of raised and Schottky source/drain MOSFETs using a novel tunneling contact model. *Technical Digest of the International Electron Devices Meeting,* (1998).

18. Janietz S., Bradley D. D. C., Grell M., Giebeler C., Inbasekaran M. & Woo E. P. Electrochemical determination of the ionization potential and electron affinity of poly(9,9-dioctylfluorene). *Appl. Phys. Lett.***73**, 2453-2455 (1998).

19. Braun S., Salaneck W. R. & Fahlman M. Energy-Level Alignment at Organic/Metal and Organic/Organic Interfaces. *Adv. Mater.* **21**, 1450-1472 (2009).

20. Hummer K, Ambrosch-Draxl C. Electronic properties of oligoacenes from first principles. *Phys. Rev. B* **72**, 205205 (2005).

21. Poplavskyy D, Nelson J, Bradley DDC. Ohmic hole injection in poly(9,9-dioctylfluorene) polymer light-emitting diodes. *Appl. Phys. Lett.***83**, 707-709 (2003).

22. Koch N, Vollmer A, Elschner A. Influence of water on the work function of conducting poly(3,4-ethylenedioxythiophene)/poly(styrenesulfonate). *Appl. Phys. Lett.***90**, 043512 (2007).

23. Nicolai HT, Wetzelaer GAH, Kuik M, Kronemeijer AJ, de Boer B, Blom PWM. Space-charge-limited hole current in poly(9,9-dioctylfluorene) diodes. *Appl. Phys. Lett.***96**, 172107 (2010).

24. van Mensfoort SLM, Vulto SIE, Janssen RAJ, Coehoorn R. Hole transport in polyfluorene-based sandwich-type devices: Quantitative analysis of the role of energetic disorder. *Phys. Rev. B* **78**, 085208 (2008).

25. Fumagalli L, Binda M, Natali D, Sampietro M, Salmoiraghi E, Di Gianvincenzo P. Dependence of the mobility on charge carrier density and electric field in poly(3-hexylthiophene) based thin film transistors: Effect of the molecular weight. *J. Appl. Phys.* **104**, 084513 (2008).

26. Hu WJ*, et al.* Universal Ferroelectric Switching Dynamics of Vinylidene Fluoride-trifluoroethylene Copolymer Films. *Sci. Rep.* **4**, 4772 (2014).

27. Landauer R, Young DR, Drougard ME. Polarization Reversal in the Barium Titanate Hysteresis Loop. *J. Appl. Phys.* **27**, 752-758 (1956).

28. Pulvari CF, Kuebler W. Phenomenological Theory of Polarization Reversal in BaTiO3 Single Crystals. *J. Appl. Phys.* **29**, 1315-1321 (1958).

29. So YW, Kim DJ, Noh TW, Yoon J-G, Song TK. Polarization switching kinetics of epitaxial Pb(Zr0.4Ti0.6)O3 thin films. *Appl. Phys. Lett.***86**, 092905 (2005).

30. Yang SM*, et al.* ac dynamics of ferroelectric domains from an investigation of the frequency dependence of hysteresis loops. *Phys. Rev. B* **82**, 174125 (2010).

31. Avrami M. Kinetics of Phase Change. I General Theory. *J. Chem. Phys.* **7**, 1103-1112 (1939).

32. Ishibashi Y, Takagi Y. Note on Ferroelectric Domain Switching. *J. Phys. Soc. Jpn.* **31**, 506-510 (1971).

33. Tagantsev AK, Cross LE, Fousek J. *Domains in ferroic crystals and thin films*, Springer (2010).

34. Zhao D, Katsouras I, Asadi K, Blom PWM, de Leeuw DM. Switching dynamics in ferroelectric P(VDF-TrFE) thin films. *Phys. Rev. B* **92**, 214115 (2015).

35. Wang D, Kopidakis N, Reese MO, Gregg BA. Treating Poly(3-hexylthiophene) with Dimethylsulfate Improves Its Photoelectrical Properties. *Chem. Mater.* **20**, 6307-6309 (2008).

36. Liang Z, Nardes A, Wang D, Berry JJ, Gregg BA. Defect Engineering in π-Conjugated Polymers. *Chem. Mater.* **21**, 4914-4919 (2009).

37. Gregg BA. Transport in Charged Defect-Rich π-Conjugated Polymers. *J. Phys. Chem. C* **113**, 5899-5901 (2009).

38. Kaake LG, Barbara PF, Zhu XY. Intrinsic Charge Trapping in Organic and Polymeric Semiconductors: A Physical Chemistry Perspective. *J. Phys. Chem. Lett.* **1**, 628-635 (2010).

39. Asadi K.*, et al.* Spinodal Decomposition of Blends of Semiconducting and Ferroelectric Polymers. *Adv. Funct. Mater.* **21**, 1887-1894 (2011).

40. Hu Z, Tian M, Nysten B, Jonas AM. Regular arrays of highly ordered ferroelectric polymer nanostructures for non-volatile low-voltage memories. *Nat. Mater.* **8**, 62-67 (2009).

41. Nougaret L*, et al.* Nanoscale Design of Multifunctional Organic Layers for Low-Power High-Density Memory Devices. *ACS Nano* **8**, 3498-3505 (2014).

42. Kassa HG*, et al.* The Ferro- to Paraelectric Curie Transition of a Strongly Confined Ferroelectric Polymer. *Macromolecules* **47**, 4711-4717 (2014).

43. Lenz T.*, et al.* Microstructured organic ferroelectric thin film capacitors by solution micromolding. *Phys. Status Solidi A* **212**, 2124-2132 (2015).

44. Lenz T.*, et al.* Downscaling and Charge Transport in Nanostructured Ferroelectric Memory Diodes Fabricated by Solution Micromolding. *Adv. Funct. Mater.* **26**, 5111-5119 (2016).

45. Torricelli F.*, et al.* Ambipolar Organic Tri-Gate Transistor for Low-Power Complementary Electronics. *Adv. Mater.* **28**, 284-290 (2016).
